# Supplementary material for: Systematic evaluation of BBB-penetrant AAV capsids in marmosets identifies VCAP-102 as a highly efficient brain-transducing capsid
Source: Mol Ther Adv. 2026 Jun 16;34(3):201783. doi: 10.1016/j.omta.2026.201783 (PMC13332002; doi:10.1016/j.omta.2026.201783)
Supplement: Document S1. Figures S1–S5 and Tables S1 and S2 [file mmc1.pdf]

OMTA, Volume *34*

Supplemental information

Systematic evaluation of BBB-penetrant AAV capsids in marmosets identifies VCAP-102 as a highly efficient brain-transducing capsid

Yasunori Matsuzaki, Ayumu Konno, Kenji Sakamoto, Yasuo Uchida, Tetsuya Terasaki, and Hirokazu Hirai

Figure S1.

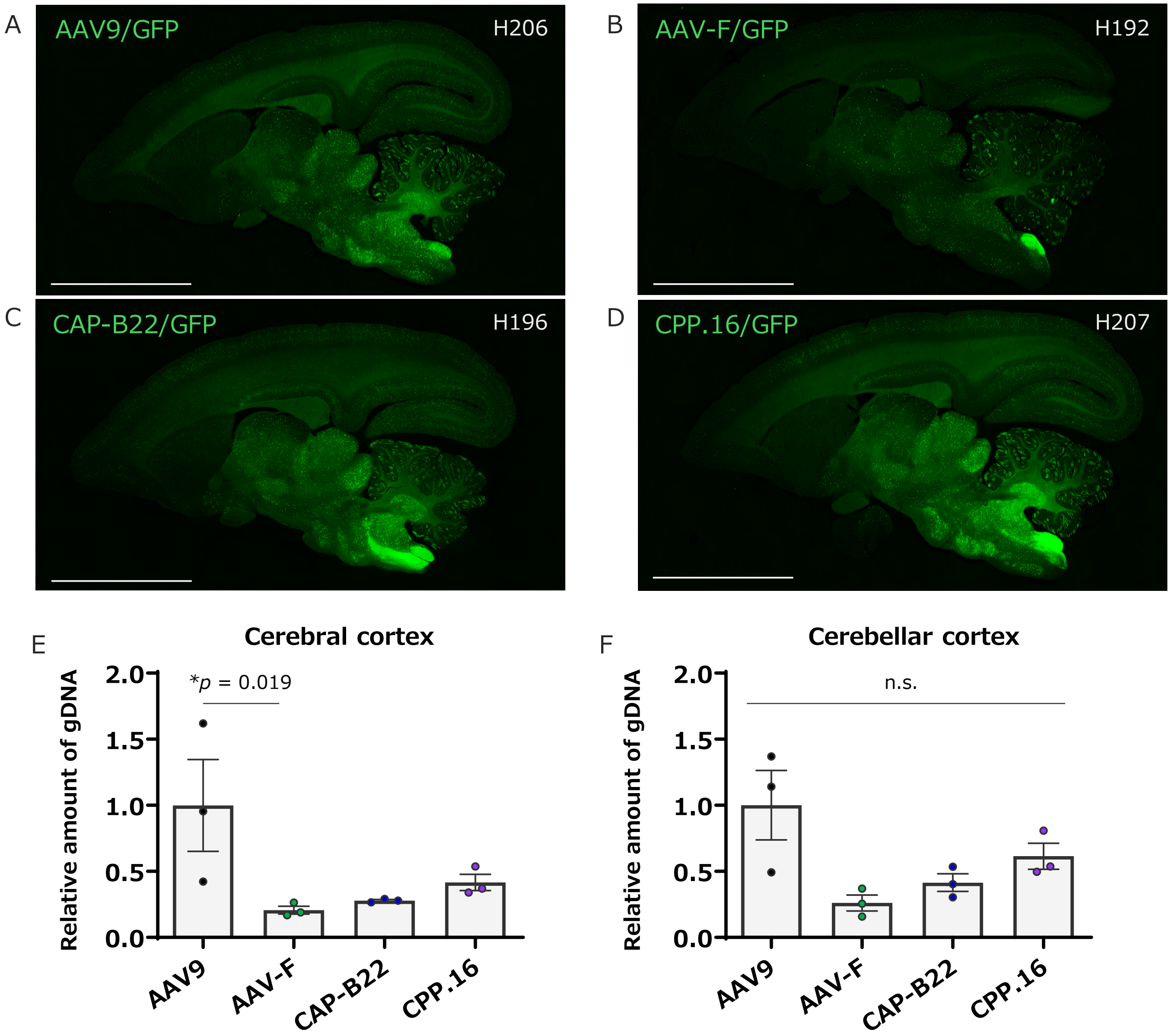

Figure S1. Quantification of AAV genome DNA levels in the marmoset brain following intravenous injection of AAV-F, CAP-B22, or CPP.16.

(A–D) Representative coronal brain images from marmosets injected with AAV9-GFP (A), AAV-F-GFP (B), CAP-B22-GFP (C), or CPP.16-GFP (D). Scale bars; 10 mm.

(E, F) Quantification of AAV genomic DNA (gDNA) in the cerebral cortex (E) and cerebellar cortex (F) by qPCR. gDNA levels for each capsid variant were normalized to those of AAV9. Consistent with the results described in the main text, none of the three variants (AAV-F, CAP-B22, CPP.16) exceeded AAV9 in cortical gDNA levels, and AAV-F showed significantly lower levels than AAV9 in the cerebral cortex ($p = 0.019$). No significant differences were observed among capsids in the cerebellar cortex. Error bars indicate the SEM, and each dot represents the value obtained from an individual marmoset.

Figure S2.

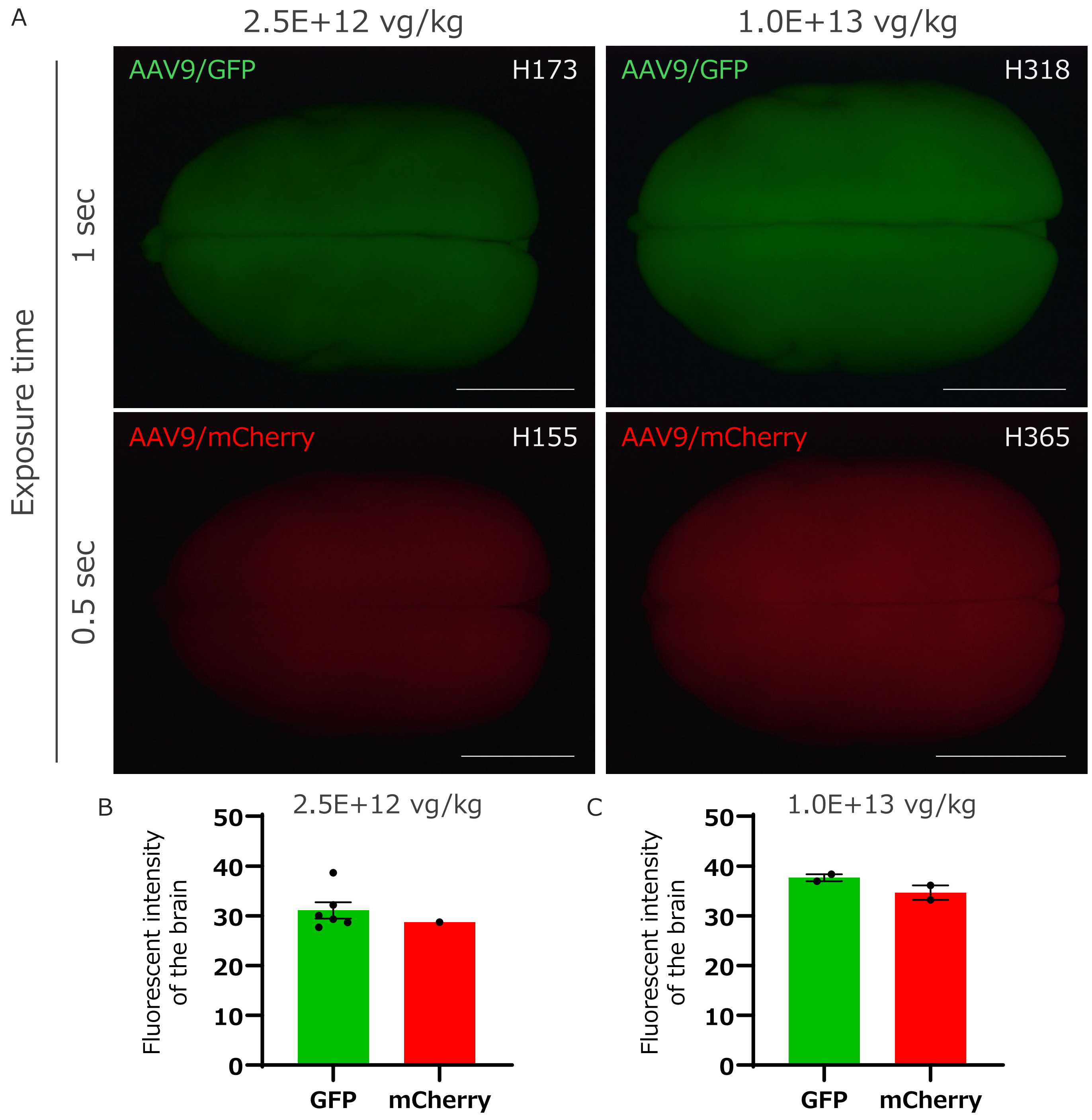

Figure S2. Comparison of whole-brain fluorescence intensity between AAV9-GFP and AAV9-mCherry at different exposure times.

(A) Whole-brain fluorescence images of marmosets intravenously injected with AAV9-GFP (1 s exposure) or AAV9-mCherry (0.5 s exposure) at doses of 2.5×10^{12} vg/kg (left) or 1.0×10^{13} vg/kg (right). Scale bars; 10 mm.

(B, C) Quantification of brain fluorescence intensity at each dose. AAV9-GFP (1 s exposure) and AAV9-mCherry (0.5 s exposure) showed comparable whole-brain fluorescence levels. Therefore, GFP and mCherry signals were imaged using 1 s and 0.5 s exposure times, respectively, throughout this study.

Figure S3.

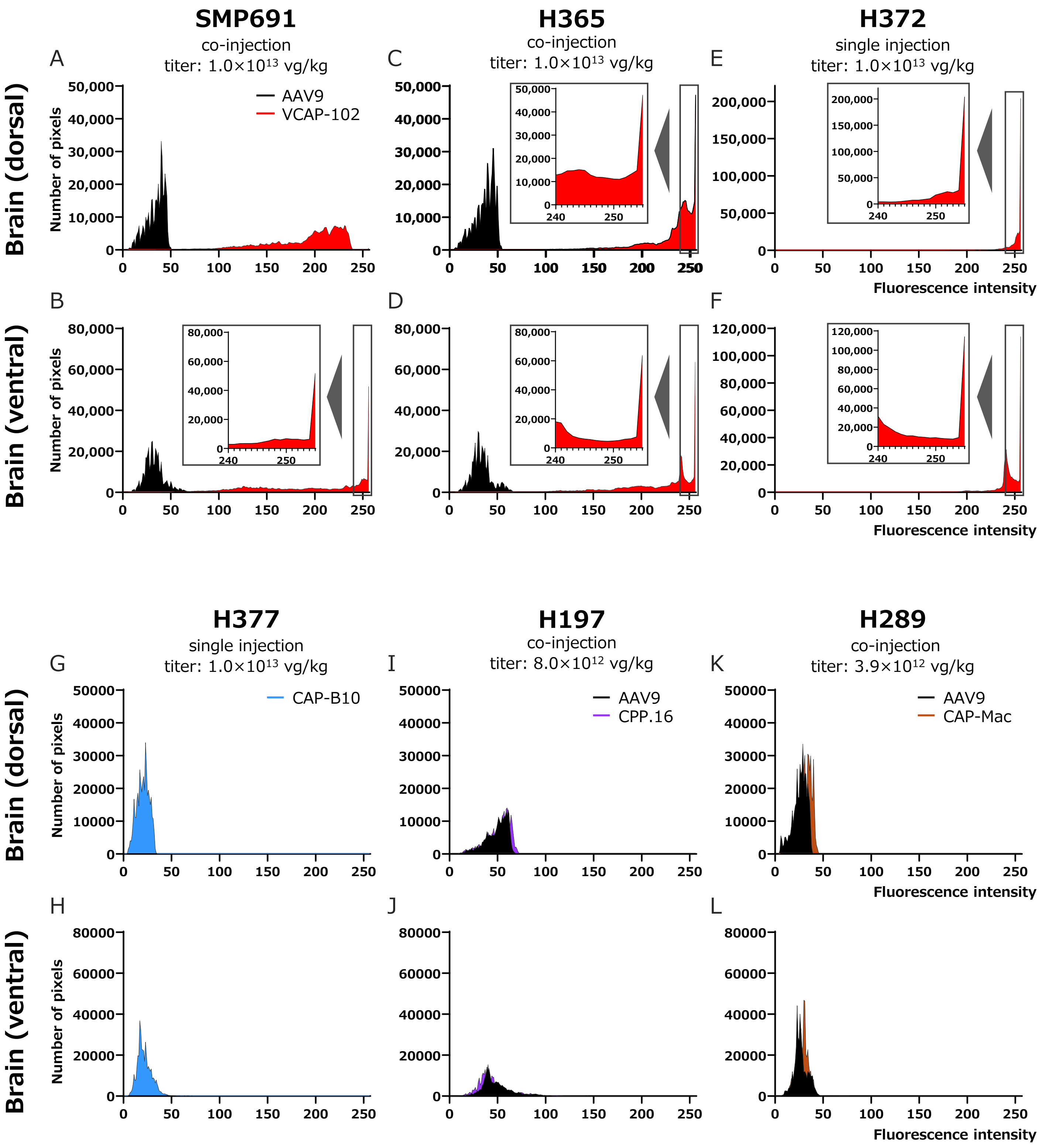
SMP691
co-injection
titer: 1.0×10^13 vg/kg
H365
co-injection
titer: 1.0×10^13 vg/kg
H372
single injection
titer: 1.0×10^13 vg/kg
A
B
C
D
E
F
Brain (dorsal)
Brain (ventral)
Number of pixels
Fluorescence intensity
AAV9
VCAP-102
H377
single injection
titer: 1.0×10^13 vg/kg
H197
co-injection
titer: 8.0×10^12 vg/kg
H289
co-injection
titer: 3.9×10^12 vg/kg
G
H
I
J
K
L
CAP-B10
AAV9
CPP.16
AAV9
CAP-Mac

Figure S3. Whole-brain fluorescence intensity histograms of AAV-injected marmosets.

Whole-brain dorsal (A, C, E, G, I, K) and ventral (B, D, F, H, J, L) fluorescence intensity histograms obtained three to four weeks after intravenous AAV administration in six marmosets (SMP691, H289, H365, H372, H377, H197). AAV9, VCAP-102, CAP-B10, CPP.16, and CAP-Mac were evaluated either by single injection or by co-injection, as indicated for each animal. Fluorescence intensity was recorded as relative values ranging from 0 to 255. For capsids other than VCAP-102, exposure times were minimized to the detection threshold (GFP: 1 s, mCherry: 0.5 s).

Insets in panels B–F show enlarged views of the upper-intensity range (240–255), highlighting the distribution of pixels near the saturation level. Because saturated pixels were recorded as 255, all brain regions in which VCAP-102 expression exceeded the dynamic range of detection appear as a vertical accumulation at this value.

Despite using shortened exposure conditions, all three marmosets injected with VCAP-102 exhibited strong GFP expression with signals saturating the upper limit (255 a.u.) across multiple brain regions. As saturated values were stored as 255 in the histogram analysis, the actual fluorescence intensity of VCAP-102 relative to AAV9 is likely higher than the ratios shown in Figure 3.

Figure S4.

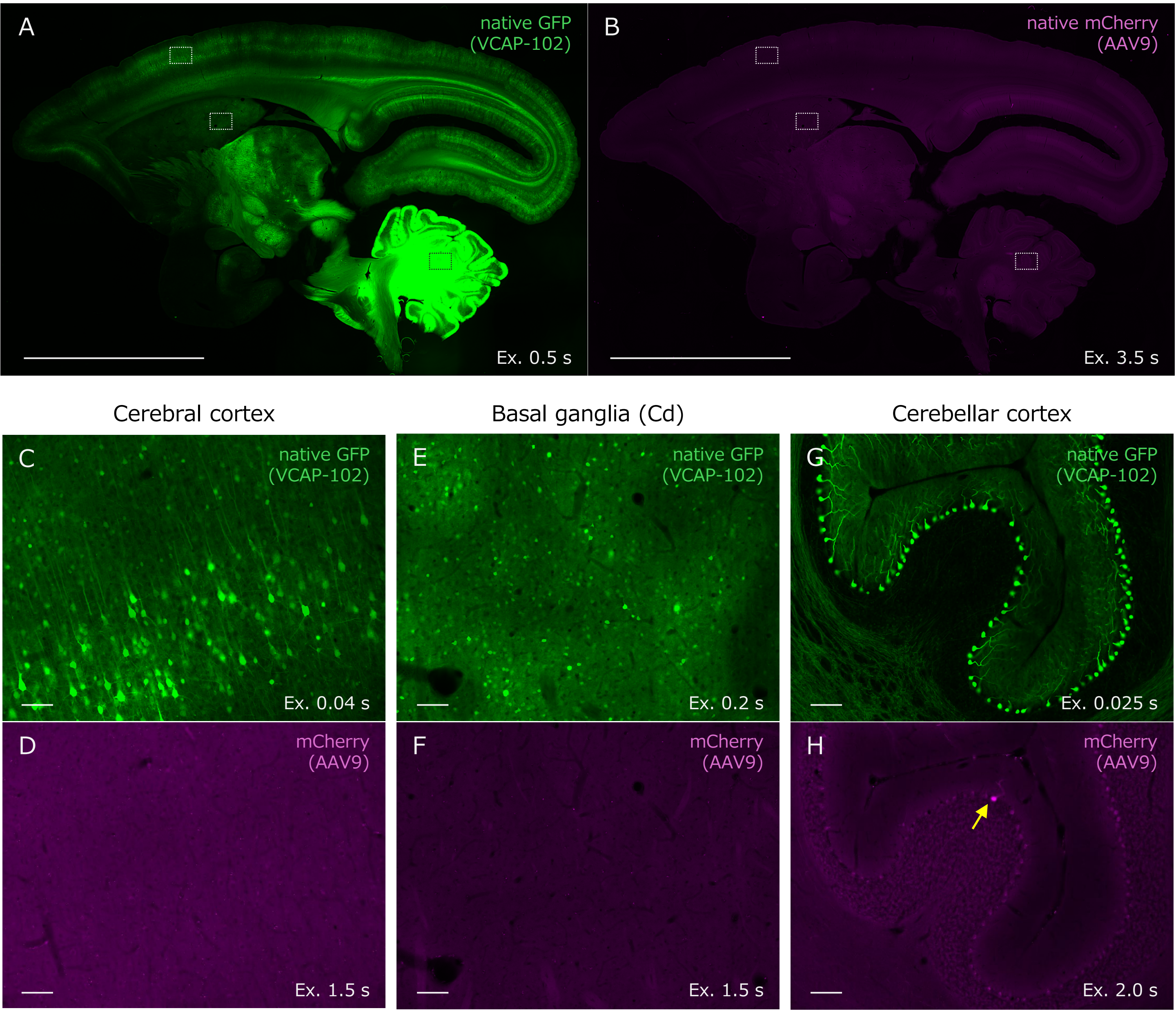

Figure S4. Intravenous administration of VCAP-102 induces widespread GFP expression throughout the brain.
(A, B) Representative sagittal brain images showing native GFP (A) and mCherry (B) fluorescence in marmoset H365 co-injected with VCAP-102-GFP and AAV9-mCherry. GFP images were acquired with an exposure time of 0.5 s (Ex. 0.5 s). Because mCherry fluorescence was much weaker, the image was acquired with a longer exposure time of 3.5 s (Ex. 3.5 s). Scale bars, 10 mm.
(C–H) Higher-magnification images of the cerebral cortex (C, D), caudate nucleus (E, F), and cerebellar cortex (G, H). Native GFP (upper panels) and mCherry (lower panels) fluorescence images are shown. These images correspond to the boxed regions in the whole sagittal sections shown in (A, B) and were acquired at the exposure times indicated in the lower right corner of each panel. GFP signals were readily detected in numerous cells across all regions, whereas mCherry signals were barely detectable even with substantially longer exposure times. Only a single labeled cell was observed in the cerebellum (arrow in panel H). Scale bars, 100 µm.

Figure S5.

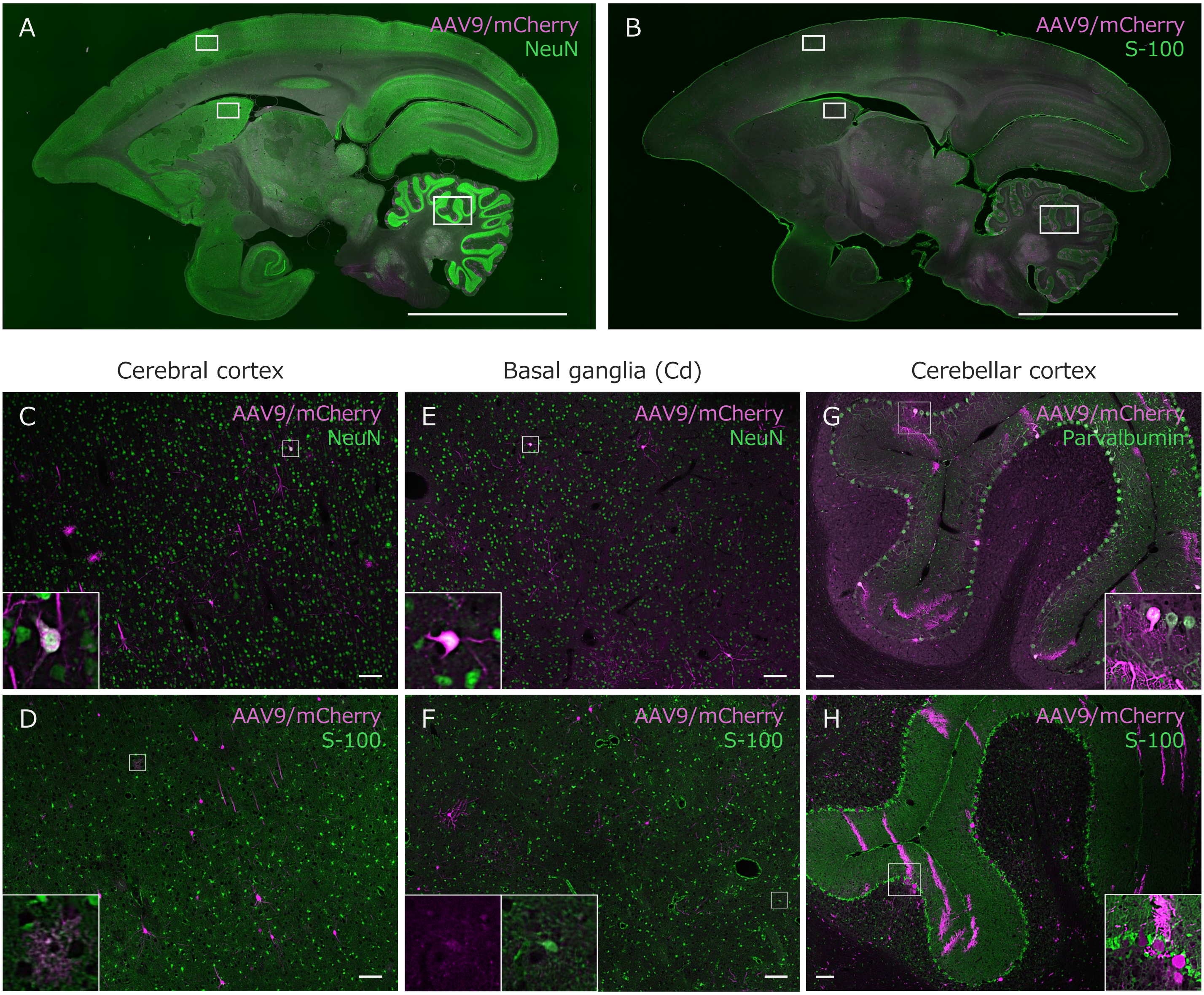

Figure S5. Representative immunohistochemical images of the brain from a marmoset intravenously injected with AAV9-mCherry.

(A, B) Immunohistochemistry of whole-brain sagittal sections showing mCherry/NeuN (A) and mCherry/S100β (B) signals in marmoset H365 injected with AAV9-mCherry. Brain sections were immunolabeled for mCherry together with NeuN (neuron marker), S100β (astrocyte marker), or parvalbumin (PV; inhibitory neuron marker). Scale bars, 10 mm.

(C–H) Higher-magnification images of the cerebral cortex (C, D), caudate nucleus (Cd; E, F), and cerebellar cortex (G, H). Panels show mCherry/NeuN (C, E), mCherry/PV (G), and mCherry/S100β (D, F, H) immunostaining. These images correspond to the boxed regions in (A, B). Insets in the lower left of each panel show further magnified views. Scale bars, 10 mm (A, B) and 100 µm (C-H).

Table S1. Summary of wild-type AAV serotypes and BBB-penetrant AAV capsid variants (2016–2025).

Name of AAV vector	AA452–458 (VR-IV)	AA587–590 (VR-VIII)	Proposal receptor	Method	Publication year
AAV9 (AAV hu.14)	NGSGQNQ	AQAQ	-	-	2004
AAV-PHP.B	NGSGQNQ	AQTLAVPFKAQ	Ly6A (mouse only)	CREATE	2016
AAV-PHP.eB	NGSGQNQ	DGTLAVPFKAQ	Ly6A (mouse only)	CREATE	2017
AAV-F	NGSGQNQ	AQFVVGQSYAQ	Ly6C1 (mouse only)	iTransduce	2019
AAV.CAP-B10	DGAATKN	DGTLAVPFKAQ	(unknown)	M-CREATE	2022
AAV.CAP-B22	DGQSSKS	DGTLAVPFKAQ	(unknown)	M-CREATE	2022
AAV.CPP.16	NGSGQNQ	AQTVSALKAQ	(transcytosis by the cpp)	CPPs-insertion	2022
AAV.CAP-Mac	NGSGQNQ	AQLNTTKPIAQ	(unknown)	*in vivo* directed evolution	2023
AAV-IR	NGSGQNQ	AQGGGSSRHDNGTKGGSGSAQ	(unknown)	TRADE	2024
AAV-Ly6H	NGSGQNQ	AQGGGSNYTKEAMSGGGGSAQ	(unknown)	TRADE	2024
VCAP-102	NGHDSPHKSGQNQ	AQAQ	ALPL/TNAP	TRACER	2025
AAV2	SGTTTQS	NRQA	-	-	1996
AAV-BR1	SGTTTQS	QRGNRGTEWDAQA	(unknown)	random AAV display peptide libraries	2016
AAV-BR1N	SGTTTQS	NRGNRGTEWDAQA	(unknown)	BR1 variant (587N)	2023

This table lists the amino acid sequences inserted into variable region IV (VR-IV; aa452–458 in AAV9) and variable region VIII (VR-VIII; aa587–590 in AAV9), along with proposed or validated endothelial receptors, engineering strategies, and publication years for each capsid. AAV9 (published in 2004) and AAV2 (1996) represent wild-type serotypes. BBB-penetrant AAV capsid variants have been reported from 2016 to 2025. BR1 is not a BBB-penetrant variant but an AAV2-based capsid that efficiently transduces brain endothelial cells; replacement of residue 587 (Q→N) generates BR1N, which acquires BBB permeability in mice.
Amino acids 452–458 are located within the VR-IV region, and amino acids 587–590 are located within the VR-VIII region. Receptor information is provided only when experimentally demonstrated or explicitly proposed in the original studies. “Method” indicates the engineering strategy used (e.g., CREATE, M-CREATE, TRACER, CPP insertion, or directed evolution). Among the listed variants, VCAP-102 is the only capsid with a confirmed endothelial receptor (ALPL/TNAP) in non-human primates.

Table S2. Individual marmoset information and whole-brain dorsal and ventral fluorescence intensities following intravenous AAV administration.

Titer (vg/kg)	Individual ID	Name	AAV capsid	Fluorescent protein	Mean fluorescence intensity dorsal/ventral (a.u., 0–255)
1.0×10^{12}	H193	Ginga	AAV9	GFP	42.2 / 40.8
			CPP.16	mCherry	51.1 / 61.1
2.5×10^{12}	H165	Nogi	AAV9	GFP	27.5 / 29.4
			PHP.eB	mCherry	28.8 / 28.4
	H184	Tanpopo	AAV9	GFP	33.5 / 27.5
			PHP.eB	mCherry	32.0 / 24.2
	H173	Waboku	AAV9	GFP	28.6 / 29.7
			AAV-F	mCherry	28.4 / 22.6
	H186	Fuuwa	AAV9	GFP	30.1 / 32.8
			CAP-B10	mCherry	31.8 / 29.5
	H187	Kaoru	AAV9	GFP	38.6 / 36.3
			CAP-B22	mCherry	29.4 / 27.6
	H183	Uguisu	AAV9	GFP	30.1 / 31.6
			CAP-B22	mCherry	28.9 / 26.2
	H154	Edamame	AAV9	GFP	29.3 / 31.9
			CPP.16	mCherry	28.9 / 27.0
	H155	Okura	AAV9	mCherry	27.7 / 27.3
			CPP.16	GFP	34.5 / 39.0
	H188	Umi	AAV9	GFP	32.2 / 33.9
			BR1N	mCherry	29.3 / 25.6
3.9×10^{12}	H289	Yoru	AAV9	GFP	25.3 / 26.1
			CAP-Mac	mCherry	30.0 / 27.7
5.0×10^{12}	H057	Kasumi	AAV9	GFP	23.2 / 22.7
	I4471	Ren	AAV9	GFP	16.2 / 19.0
	H058	Mitsuba	PHP.B	GFP	22.4 / 26.2
	I4466	Suigetsu	PHP.B	GFP	20.4 / 22.4
8.0×10^{12}	H197	Kuri	AAV9	GFP	47.9 / 50.1
			CPP.16	mCherry	49.9 / 44.4
1.0×10^{13}	H137	Touga	PHP.eB	GFP	18.7 / 25.1
	H148	Tougetsu	PHP.eB	GFP	16.5 / 21.5
	H377	Bon	CAP-B10	GFP	19.4 / 21.1
	H318	Serori	AAV9	GFP	36.9 / 39.2
			AAV-IR	mCherry	32.1 / 29.4
	H319	Paseri	AAV9	GFP	38.4 / 42.0
			AAV-Ly6H	mCherry	44.8 / 43.1
	SMP691	Kinmokusei	AAV9	mCherry	33.2 / 32.1
			VCAP-102	GFP	188.8 / 187.1
	H365	Nodoka	AAV9	mCherry	36.1 / 32.8
			VCAP-102	GFP	229.4 / 213.2
	H372	Nana	VCAP-102	GFP	248.6 / 241.4
2.0×10^{13}	H140	Sekka	AAV9	GFP	21.6 / 22.6
	H141	Neyuki	PHP.eB	GFP	35.9 / 41.3

Mean pixel intensity (0–255 a.u.) was quantified on the dorsal ventral surface of the whole brain approximately 4 weeks after intravenous delivery of each AAV vector. For animals receiving co-injections, fluorescence from AAV9 (GFP or mCherry) serves as the internal control for the paired variant. The table lists, for each individual: AAV dose (vg/kg), animal ID, name, capsid, expressed fluorophore, and mean dorsal and ventral fluorescence intensity. Measurements were performed under standardized exposure settings (GFP: 1 s; mCherry: 0.5 s).
