# Supplementary material for: Systematic evaluation of BBB-penetrant AAV capsids in marmosets identifies VCAP-102 as a highly efficient brain-transducing capsid
Source: Mol Ther Adv. 2026 Jun 16;34(3):201783. doi: 10.1016/j.omta.2026.201783 (PMC13332002; doi:10.1016/j.omta.2026.201783)
Supplement: Document S2. Article plus supplemental information [file mmc2.pdf]

Molecular Therapy
Advances
Original Article

Systematic evaluation of BBB-penetrant AAV capsids in marmosets identifies VCAP-102 as a highly efficient brain-transducing capsid

Yasunori Matsuzaki,[1,2] Ayumu Konno,[1,2] Kenji Sakamoto,[1] Yasuo Uchida,[3] Tetsuya Terasaki,[4] and Hirokazu Hirai[1,2]

[1]Department of Neurophysiology & Neural Repair, Gunma University Graduate School of Medicine, Maebashi, Gunma 371-8511, Japan; [2]Viral Vector Core, Gunma University, Initiative for Advanced Research, Maebashi, Gunma 371-8511, Japan; [3]Department of Molecular Systems Pharmaceutics, Graduate School of Biomedical and Health Science, Hiroshima University, Higashihiroshima, Hiroshima 739-8511, Japan; [4]School of Pharmacy, University of Eastern Finland, 70211 Kuopio, Finland

Recent advances in engineering adeno-associated virus (AAV) capsids capable of crossing the blood-brain barrier (BBB) have led to the development of numerous variants aimed at enhancing central nervous system gene delivery. Over the past decade, as new BBB-penetrant capsids were reported or independently identified through our own capsid library screening, we systematically evaluated their performance in adult marmosets, using a standardized experimental framework. Here, we present a cross-comparison of eleven AAV variants, predominantly AAV9-derived, alongside native AAV9 following systemic administration. Whole-brain reporter expression was quantitatively assessed using uniform imaging and analysis pipelines to enable relative comparison across animals. Under these conditions, most previously reported and newly identified BBB-penetrant variants—including those obtained through our own screening efforts—did not exhibit brain transduction levels clearly distinguishable from those of AAV9 based on whole-brain fluorescence intensity. In contrast, VCAP-102 consistently produced markedly higher brain-wide reporter expression across animals, accompanied by substantially increased vector genome copy numbers in the marmoset cortex, with most GFP-positive cells corresponding to neurons. These findings provide a systematic benchmark for evaluating BBB-penetrant AAV capsids in non-human primates and identify VCAP-102 as a highly efficient vector for systemic brain gene delivery.

INTRODUCTION

In humans, the brain is the second heaviest organ after the liver and requires a continuous supply of oxygen and glucose delivered through an extensive vascular network. The blood-brain barrier (BBB) tightly regulates molecular exchange between the circulation and the brain parenchyma. Efficient gene delivery across the BBB is, therefore, essential for developing transformative therapies for neurological diseases with widespread pathology, such as Alzheimer disease and inherited neurodegenerative disorders.

Among naturally occurring serotypes, adeno-associated virus 9 (AAV9) has been shown to cross the BBB. However, even high-dose recombinant AAV9 administered intravenously results in <1% gene transduction in the mouse brain. In 2016, Gradinaru and colleagues developed PHP.B by inserting a 7-amino acid peptide into the AAV9 variable region VIII (VR-VIII) using the CREATE platform, demonstrating exceptional BBB penetration in C57BL/6J mice.[1] Yet, our evaluation revealed that PHP.B did not improve brain transduction in marmosets[2]—a result not previously reported, highlighting a pronounced species barrier. PHP.B was later shown to rely on Ly6A,[3,4] a receptor not expressed in primates, thereby limiting its translational potential.

Following this, whenever a new BBB-penetrant capsid was described, we obtained its sequence upon publication, produced the corresponding vector, and evaluated it side-by-side in our standardized marmoset intravenous administration system. Although prior reports have described enhanced BBB transduction efficiency for CAP-B22, CAP-B10,[5,6] CPP.16,[7] and CAP-Mac[8] relative to AAV9, our side-by-side evaluation under identical dosing and analytical conditions in adult marmosets did not reveal a pronounced difference in overall whole-brain reporter expression compared with AAV9.

In 2024, Voyager Therapeutics identified 13 candidate VR-IV insertion sequences, using the TRACER platform.[9] Among them, VCAP-102, containing the HDSPHKSG insertion, was reported in 2025 to show robust BBB penetration in mice and non-human primates

Received 5 January 2026; accepted 12 June 2026;
https://doi.org/10.1016/j.omta.2026.201783.

Correspondence: Ayumu Konno, Department of Neurophysiology & Neural Repair, Gunma University Graduate School of Medicine, Maebashi, Gunma 371-8511, Japan.
E-mail: konnoa@gunma-u.ac.jp
Correspondence: Hirokazu Hirai, Department of Neurophysiology & Neural Repair, Gunma University Graduate School of Medicine, Maebashi, Gunma 371-8511, Japan.
E-mail: hirai@gunma-u.ac.jp

© 2026 The Author(s). Published by Elsevier Inc. on behalf of The American Society of Gene and Cell Therapy.
This is an open access article under the CC BY license (http://creativecommons.org/licenses/by/4.0/).

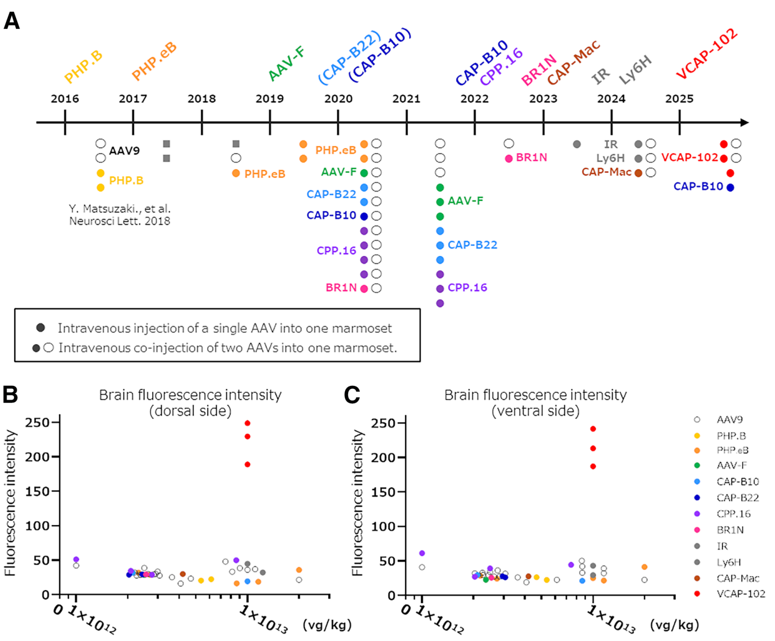

Figure 1. Decade-long systematic evaluation of BBB-penetrant AAV capsid variants in marmosets over the past decade

(A) Timeline summarizing BBB-penetrant AAV capsid variants reported between 2016 and 2025 and evaluated in our marmoset intravenous injection system. Each newly reported AAV capsid (e.g., PHP.B, PHP.eB, AAV-F, CAP-B22, CAP-B10, CPP.16, BR1N, CAP-Mac, and VCAP-102) was produced upon publication and assessed under standardized experimental conditions. CAP-B22 and CAP-B10 were reported in 2020; however, because they were available only as preprints, they are indicated in parentheses. A single circle (or square) indicates one marmoset that received an intravenous injection of either AAV9 or a single variant capsid. Pairs of adjacent circles represent one marmoset that was intravenously injected with a mixture of AAV9 and a variant capsid. The gray squares in 2017–2018 indicate the marmosets used for our screening based on a modified CREATE method. The gray circles in 2023–2024 represent the marmosets that received intravenous administration of AAV9 capsid variants targeting the insulin receptor (IR) or Ly6H. (B and C) Relative brain-wide fluorescence intensity (0–255 a.u.) measured 4 weeks after intravenous AAV administration. (B) Dorsal view and (C) ventral view of whole brains from marmosets injected with each capsid variant. Fluorescence intensities for AAV9 and each variant capsid are plotted as a function of viral dose (vg/kg). Each data point represents an individual marmoset. VCAP-102 produced the highest brain-wide fluorescence intensity among the variants evaluated.

(NHPs).[10] When tested in our established marmoset system, VCAP-102 produced markedly stronger and more widespread whole-brain transduction than AAV9 in all three animals examined.

Here, we present a decade-long systematic evaluation of previously reported and newly identified BBB-penetrant AAV capsid variants, including those derived from our own capsid library screenings, in marmosets and demonstrate that VCAP-102 is the first variant to achieve outstanding BBB permeability in this primate species.

RESULTS

AAV capsid variants reported to efficiently cross the BBB in mice

BBB-penetrant AAV capsid variants reported since 2016 and subsequently evaluated in our marmoset system are summarized in Figure 1A and Table S1.

To systematically compare BBB permeability across capsids, AAV9 or capsid variants expressing enhanced green fluorescent protein (GFP) under the control of the CBh promoter (AAV9/GFP) were intravenously administered via the femoral vein in adult marmosets. Four weeks after the injection, the animals were perfusion fixed, and whole-brain GFP fluorescence was imaged from both dorsal and ventral aspects. BBB permeability of each capsid was evaluated by comparing the fluorescence intensity of variant-injected brains with that of AAV9-injected brains under identical imaging conditions.

In 2016, Gradinaru et al. identified PHP.B—an AAV9 variant carrying a 7-amino acid insertion in VR-VIII—using the CREATE platform, demonstrating exceptional BBB permeability in C57BL/6J mice.[1] We immediately tested PHP.B in marmosets by intravenously administering PHP.B- or AAV9/GFP but observed no enhancement in brain transduction at 4 weeks.[2] We further showed that PHP.B fails to outperform AAV9 in several mouse strains, including BALB/c.[11] Later studies revealed that PHP.B utilizes Ly6A as its endothelial receptor, and strains lacking endothelial Ly6A expression do not exhibit BBB permeability.[3,4]

Subsequent capsids reported to cross the BBB in mice—including PHP.eB,[12] AAV-F (which penetrates the BBB in both C57BL/6 and BALB/c),[13] and the AAV2-based variant BR1N[14]—were also examined in our marmoset system. None of them exhibited BBB permeability appreciably greater than AAV9 (Figures 1B and 1C; Table S2).

AAV capsid variants reported to cross the BBB in NHPs

Since 2020, several capsids reported to penetrate the BBB in NHPs—including CAP-B22, CAP-B10,[5,6] CPP.16,[7] and CAP-Mac[8]—have been successively developed (Table S1). For each newly reported capsid, we rapidly produced the corresponding AAV vector and tested its efficacy following intravenous administration in marmosets (Figure 1A). Whole-brain GFP fluorescence showed no notable improvement relative to AAV9 for any variant (Figures 1B and 1C; Table S2).

We further quantified AAV genomic DNA (gDNA) in the cerebral cortex and cerebellum for AAV-F, CAP-B22, and CPP.16. The gDNA levels were comparable to AAV9 for all variants, and cortical levels were significantly lower for AAV-F (Figure S1).

Independent engineering of AAV9 variants by our group

To improve BBB permeability in marmosets, we conducted two independent engineering efforts.

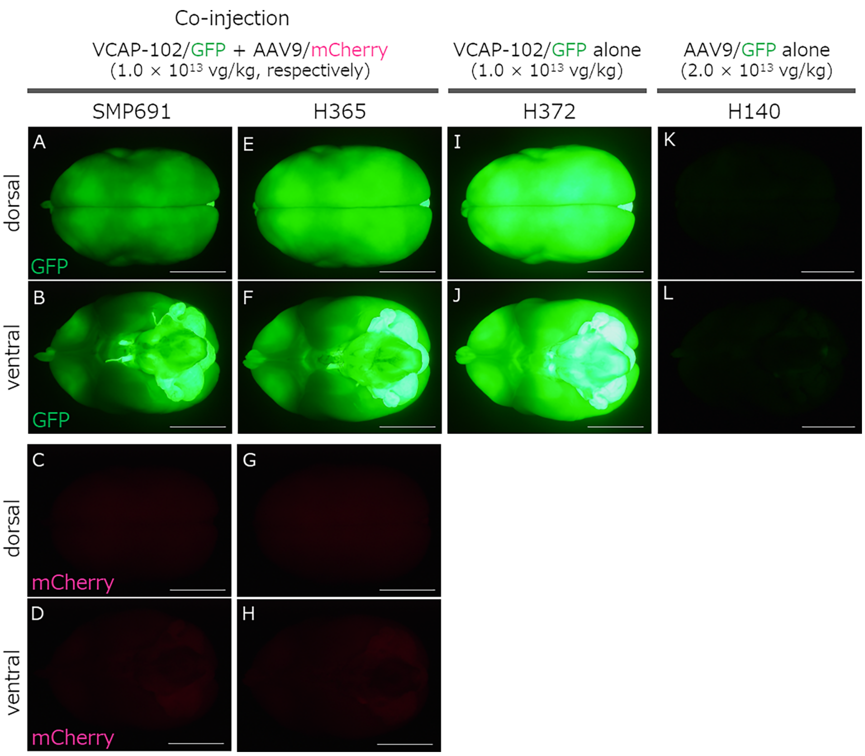

Figure 2. Brain-wide reporter expression following systemic delivery of VCAP-102 in marmosets

(A–L) Whole-brain fluorescence imaging of marmosets four weeks after intravenous administration of VCAP-102 vectors. Two marmosets (SMP691 and H365) received co-injection of VCAP-102/GFP and AAV9/mCherry at equal doses (1.0×10^{13} vg/kg each). GFP (A, B, E, and F) and mCherry (C, D, G, and H) fluorescence images are shown for dorsal and ventral views. (I–J) A third marmoset (H372) received VCAP-102/GFP alone (1.0×10^{13} vg/kg). (K and L) Brain from a marmoset (H140) injected with AAV9/GFP alone (2.0×10^{13} vg/kg) is shown as a reference control for GFP fluorescence intensity.
Scale bars, 10 mm. To enable comparison of fluorescence intensities, GFP and mCherry signals were imaged using exposure times of 1 and 0.5 s, respectively (see Figure S2).

In 2017–2018, we adapted the CREATE method[1] for *in vivo* screening in marmosets, using an AAV9 capsid library containing random 7-amino acid insertions in VR-VIII. Following intravenous administration and subsequent Cre-dependent recovery, no enriched variants demonstrating enhanced brain transduction were identified (gray squares in Figure 1A).

In 2023–2024, based on proteomic analyses identifying insulin receptor (IR) and Ly6H as enriched endothelial membrane proteins in marmosets,[15] we generated HEK293T cell lines stably expressing these targets and screened AAV9 insertion libraries for increased infectivity. Sixteen candidate variants were initially selected *in vitro* and subsequently narrowed down to two lead variants, AAV-IR and AAV-Ly6H (Figure 1). The 8-amino acid peptide sequences with GS-linkers that were inserted into these variants are listed in Table S1.

To evaluate BBB penetration *in vivo*, each capsid variant expressing mCherry was intravenously administered to adult marmosets at a vector dose of 1×10^{13} vg/kg, together with AAV9/GFP, at equal genome copy numbers. Whole-brain fluorescence imaging revealed that neither AAV-IR nor AAV-Ly6H clearly increased reporter expression relative to AAV9. Quantitative analysis of whole-brain fluorescence intensity showed values comparable to AAV9 (set to 1.0): dorsal side, AAV-IR 0.87 and AAV-Ly6H 1.17; ventral side, AAV-IR 0.75 and AAV-Ly6H 1.03. These results indicate that the IR- and Ly6H-targeting variants did not confer detectable improvements in BBB permeability in adult marmosets under the experimental conditions used in this study.

Robust and reproducible brain-wide reporter expression following systemic delivery of VCAP-102

In 2025, Nonnenmacher et al. reported VCAP-102, an AAV9-derived variant carrying the HDSPHKSG insertion in VR-IV, which efficiently crosses the BBB in both mice and NHPs.[10] We generated a VCAP-102 vector expressing GFP under the control of the CBh promoter (VCAP-102/GFP) and evaluated its performance in three independent marmosets. In two animals, VCAP-102/GFP was co-administered with an equal dose (1.0×10^{13} vg/kg) of AAV9 expressing mCherry (AAV9/mCherry), whereas the third animal received VCAP-102/GFP alone.

Four weeks after intravenous administration, whole-brain fluorescence imaging revealed consistently strong and widespread GFP expression across all three animals—markedly exceeding that observed with AAV9 and all previously evaluated variants (Figures 1 and 2). As shown in Figure 2, GFP and mCherry fluorescence were imaged using exposure times of 1 and 0.5 s, respectively, to enable comparison of fluorescence intensities (see Figure S2). Furthermore, for comparison, a marmoset injected with GFP-expressing AAV9 alone is shown on the far right of Figure 2. Notably, AAV9 was administered at twice the vector dose of VCAP-102 to provide a conservative benchmark for comparison.

Direct comparison with AAV9 by co-administration in the same animal

Importantly, all capsid variants evaluated in this study were tested under identical experimental conditions and analyzed using the same imaging pipeline, enabling standardized comparison of their relative performance in marmosets. To minimize inter-animal variability and allow direct comparison of capsid performance within the same animal, we performed co-administration experiments in which each capsid variant and AAV9 expressed different fluorescent

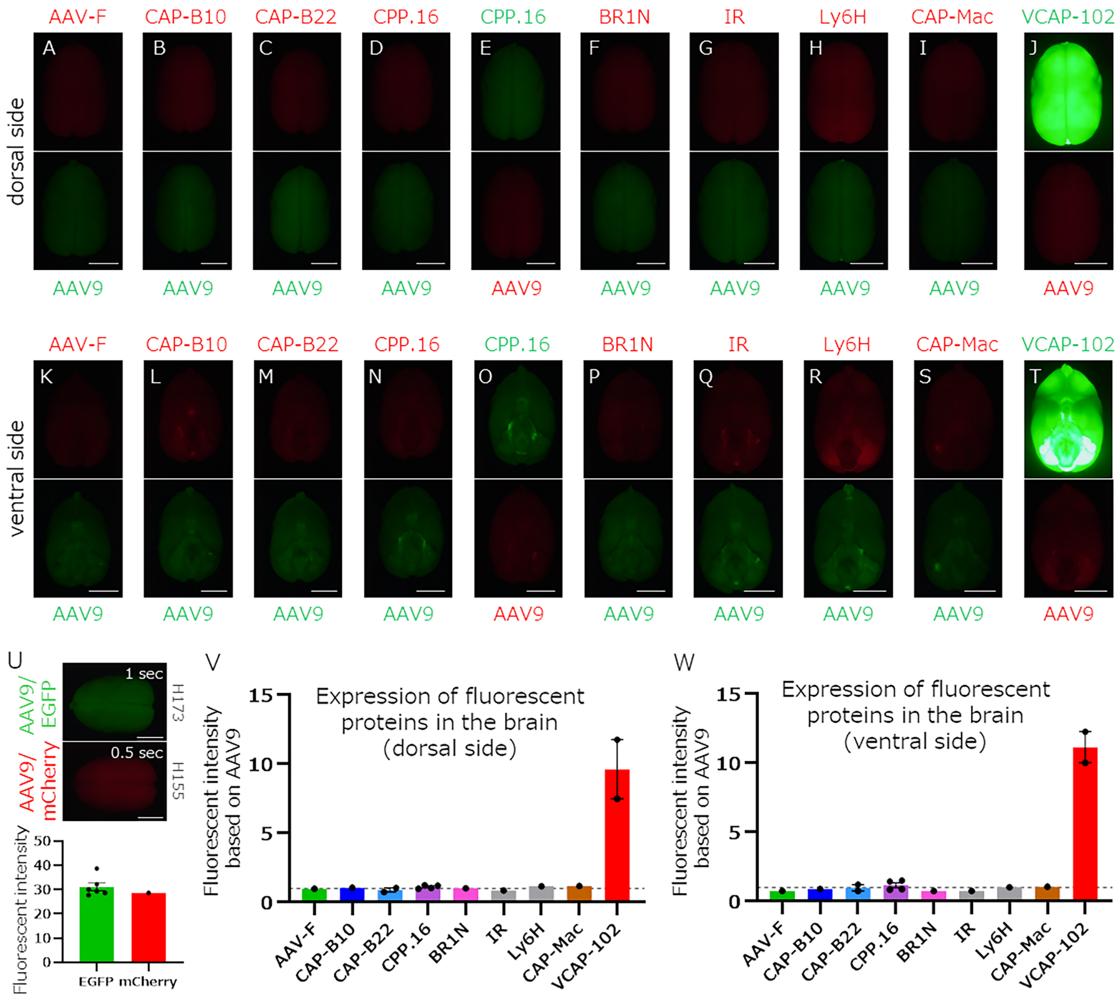

Figure 3. Co-injection assay comparing BBB permeability of AAV capsid variants with AAV9 in marmosets
(A–T) Representative dorsal (A–J) and ventral (K–T) whole-brain fluorescence images obtained 4 weeks after intravenous co-administration of each AAV capsid variant (expressing mCherry) together with AAV9 (expressing GFP). For CPP.16, the reciprocal fluorophore combination (CPP.16/GFP with AAV9/mCherry) was also evaluated (E and O). For VCAP-102, the fluorophore pairing was reversed (VCAP-102/GFP with AAV9/mCherry) to enable direct comparison with AAV9 using the same GFP reporter, consistent with single-vector injections in which all capsids were evaluated using GFP across animals (J and T). Scale bars: 10 mm. (U) Control experiment showing that AAV9/GFP (1-s exposure) and AAV9/mCherry (0.5-s exposure) produce comparable whole-brain fluorescence levels, validating the exposure settings used throughout the study. (V and W) Fluorescence intensity ratios of each variant relative to co-administered AAV9 in the same animal, calculated separately for the dorsal (V) and ventral (W) brain surfaces. All capsid variants except VCAP-102 exhibited fluorescence ratios near 1, indicating that BBB permeability was not clearly distinguishable from that of AAV9 using this approach. In contrast, VCAP-102 showed >10-fold higher fluorescence than AAV9 in both animals tested, confirming its uniquely strong BBB penetration in marmosets.

reporters. Both vectors were administered at equal genome copy numbers (vg/kg) for each capsid comparison.

In most experiments, the capsid variants expressed mCherry, whereas the control AAV9 expressed GFP. For VCAP-102, the fluorophores were reversed (VCAP-102/GFP and AAV9/mCherry) to facilitate visualization of the strong reporter signal produced by this capsid. In addition, for CPP.16, experiments were performed using both reporter configurations to exclude potential reporter-dependent bias. This dual-reporter strategy enabled direct within-animal comparison of the brain transduction efficiency between each variant and AAV9 following systemic administration (Figures 3A–3T). This within-animal comparison strategy minimizes inter-animal variability and enables a more rigorous evaluation of relative capsid performance in NHPs.

When AAV9/GFP and AAV9/mCherry were administered under identical conditions, whole-brain fluorescence levels were comparable between GFP (1-s exposure) and mCherry (0.5-s exposure) (Figures 3U and S2). These results confirmed that the two reporters produce comparable whole-brain fluorescence signals under the imaging conditions used in this study. Accordingly, GFP and mCherry

images were acquired at 1 and 0.5 s exposure times, respectively, throughout all experiments to allow direct comparison of the fluorescence intensity between capsids.

Using the corresponding whole-brain dorsal (or ventral) fluorescence intensity from AAV9-injected animals as the reference, we calculated variant-to-AAV9 fluorescence ratios. All variants except VCAP-102 exhibited ratios near 1 (Figures 3V and 3W), indicating that BBB permeability was not clearly distinguishable from that of AAV9 using this approach. In contrast, the VCAP-102/AAV9 fluorescence ratio exceeded 10-fold in both co-injected animals (rightmost bars in Figures 3V and 3W).

Fluorescence values were recorded on a relative scale of 0–255. Importantly, the exposure times used in this study—1 s for GFP and 0.5 s for mCherry—represent the lowest settings at which both fluorophores remain simultaneously detectable. Further reduction of exposure time causes the mCherry signal to fall below the detection threshold, while GFP remains readily detectable, indicating that the current settings represent the practical lower limit for dual-channel imaging.

Even under these minimum-detection exposure conditions, brain regions transduced by VCAP-102 displayed clear GFP saturation (insets in Figures S3B–3F). Thus, reducing exposure time to avoid GFP saturation would completely eliminate detectable mCherry signal, preventing a valid comparison within the same animal. Accordingly, the true fluorescence intensity of VCAP-102 relative to AAV9 is likely markedly underestimated and may greatly exceed the ratios shown in Figures 3V and 3W.

Predominant neuronal transduction following intravenous administration of VCAP-102

To evaluate the regional distribution of GFP expression in the brain, sagittal brain sections were prepared from marmosets intravenously injected with VCAP-102. GFP-positive cells were widely distributed across multiple brain regions, including the cerebral cortex, basal ganglia, and cerebellum, with particularly strong expression observed in the cerebellum (Figures S4A, S4C, S4E, and S4G). In contrast, in marmosets intravenously injected with AAV9, mCherry-labeled cells were barely detectable throughout the brain, even with prolonged exposure times (Figures S4B, S4D, S4F, and S4H).

Next, to identify the cell types transduced by VCAP-102, double immunostaining was performed using antibodies against GFP and NeuN (neuronal marker), GFP and S100β (astrocyte marker), or GFP and parvalbumin (PV; inhibitory neuron marker) (Figure 4). Similarly, the cell types transduced by AAV9 were analyzed after amplification of mCherry signals by immunostaining (Figure S5).

Higher-magnification images of the cerebral cortex and basal ganglia showed that the vast majority of VCAP-102-derived GFP-positive cells were NeuN-positive neurons, whereas only a small fraction corresponded to S100β-positive astrocytes (Figures 4C–4F). In contrast, although AAV9-derived mCherry-positive cells were much less abundant overall, both NeuN-positive neurons and S100β-positive astrocytes were observed among the transduced cells (Figures S5C–S5F).

In the cerebellar cortex, both VCAP-102-derived GFP-positive cells and AAV9-derived mCherry-positive cells were almost exclusively PV-positive Purkinje cells, characterized by large aligned cell bodies and highly developed dendritic arbors (Figures 4G, 4H, S5G, and S5H).

Quantitative analysis revealed that, in the cerebral cortex and basal ganglia, 93.3% (1,134/1,218 cells) and 95.3% (1,802/1,893 cells), respectively, of VCAP-102-derived GFP-positive cells were neurons (n = 3 animals), whereas only a small fraction were astrocytes (20/1,218 cells in the cerebral cortex and 38/1,893 cells in the basal ganglia). In contrast, among AAV9-derived mCherry-positive cells, approximately 33%–60% were neurons (17/28 cells in the cerebral cortex and 6/18 cells in the basal ganglia; n = 2 animals), whereas 20%–30% were astrocytes (6/28 and 5/18 cells, respectively) (Figures 4I and 4J). In the cerebellar cortex, both VCAP-102 and AAV9 predominantly transduced Purkinje cells (376/377 and 103/105 cells, respectively) (Figure 4K).

Together, these results demonstrate that VCAP-102 crosses the BBB more efficiently than AAV9 following intravenous administration and predominantly drives transgene expression in neurons, including Purkinje cells in the cerebellar cortex.

A VCAP-102 shows markedly higher vector genome copy numbers in the marmoset cerebral cortex than AAV9

To quantify the amount of vector delivered to the brain following intravenous administration, gDNA was extracted from the motor cortex of marmosets and analyzed by quantitative PCR (qPCR) (Figure 5A). Two animals (SMP691 and H365) received co-injection of VCAP-102/GFP and AAV9/mCherry at equal doses (1.0×10^{13} vg/kg each), whereas one animal (H372) received VCAP-102/GFP alone (1.0×10^{13} vg/kg).

Primers specific for GFP and mCherry were used to quantify vector genome (vg) copy numbers, which are presented as vg copies normalized to input gDNA (vg per 100 ng gDNA) (Figure 5B). This normalization enables direct comparison across samples without assumptions regarding cell density in heterogeneous brain tissue. In the motor cortex of SMP691 and H365, the vg copy numbers derived from VCAP-102 were 48- and 79.9-fold higher, respectively, than those derived from AAV9.

These results further support the superior brain transduction efficiency of VCAP-102 compared with AAV9 following systemic administration under identical experimental conditions in adult marmosets.

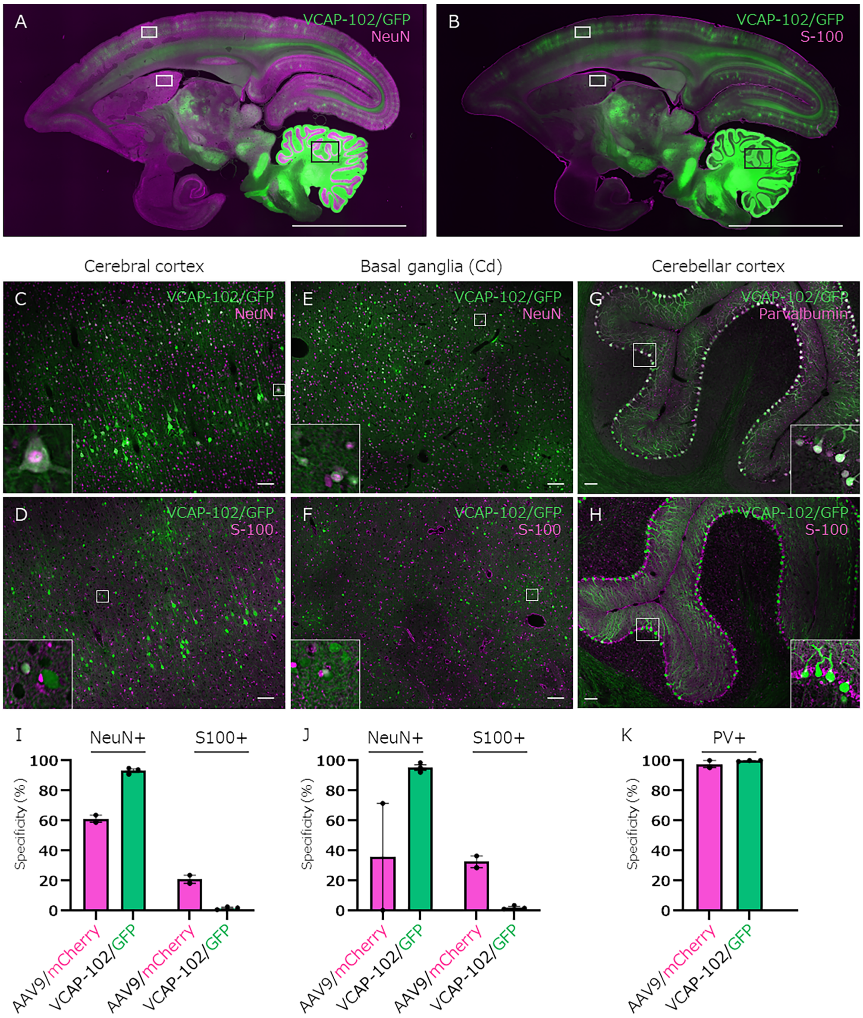

Figure 4. Predominantly neuronal GFP expression in marmosets following intravenous administration of VCAP-102

(A and B) Immunohistochemistry of whole-brain sagittal sections showing GFP/NeuN (A) and GFP/S100β (B) signals in marmoset H365 intravenously injected with VCAP-102-GFP. Brain sections were immunolabeled for GFP together with NeuN (neuron marker), S100β (astrocyte marker), or parvalbumin (PV; inhibitory neuron marker). Scale bars, 10 mm. (C–H) Higher-magnification images of the cerebral cortex (C and D), caudate nucleus (Cd; E and F), and cerebellar cortex (G and H). Shown are the immunostaining images for GFP/NeuN (C and E), GFP/PV (G), and GFP/S100β (D, F, and H). These images correspond to the boxed regions in (A) and (B). Insets in the lower left of (C)–(H) show further magnified views. Across cortical and striatal regions, the vast majority of GFP-positive cells are NeuN-positive neurons, with only a minor fraction co-labeled with S100β. In the cerebellar cortex, GFP expression is almost exclusively restricted to PV-positive Purkinje cells. Scale bars, 100 μm. (I–K) Quantitative analysis of the cell type composition among transduced cells in the cerebral cortex (I), caudate nucleus (J), and cerebellum (K) following intravenous administration of AAV9 and/or VCAP-102. VCAP-102 shows a strong neuronal bias, with ~95% of GFP-positive cells identified as neurons and only a small fraction as astrocytes, whereas AAV9 transduces both neurons and astrocytes with lower neuronal selectivity. Data for VCAP-102 were obtained from three animals (SM691, H365, and H372), and data for AAV9 were obtained from two animals (SM691 and H365). Representative immunohistochemical images of brains from marmosets injected with AAV9/mCherry are shown in Figure S5.

DISCUSSION

In this study, we present the results of a systematic, decade-long evaluation of BBB-targeted AAV capsid variants in adult marmosets under a unified experimental framework. Beginning with the initial report of the BBB-penetrant variant PHP.B in 2016,[1] we assessed newly reported capsids by generating corresponding AAV vectors expressing fluorescent reporters under the CBh promoter and testing their ability to deliver genes to the marmoset brain following intravenous administration. To our knowledge, this represents the first systematic cross-comparison of multiple BBB-targeted AAV variants in NHPs, using a unified experimental framework.

In parallel with the systematic evaluation of previously reported variants, we also undertook our own capsid engineering efforts, including *in vivo* CREATE-based screening and endothelial-targeted *in vitro* selection. Despite these efforts, no variants consistently outperformed AAV9 in adult marmosets.

Through this combined strategy of systematic evaluation and capsid engineering, we ultimately identified VCAP-102 as a capsid variant that achieves markedly enhanced BBB penetration and robust whole-brain transduction in adult marmosets. These findings highlight the importance of systematic evaluation of BBB-penetrant AAV capsids in NHPs for identifying vectors with strong translational potential for human brain gene delivery.

VCAP-102 has been reported to achieve exceptionally high levels of viral RNA in the brain, approximately 280- to 500-fold higher than that of AAV9.[10] The robust whole-brain GFP expression observed in this study is consistent with these findings and is further supported by vg quantification, which demonstrated markedly higher VCAP-102 genome copy numbers in the marmoset cortex compared with AAV9 (Figure 5).

In contrast, although previous studies have reported that CAP-B10 and CAP-B22 achieve approximately 5- to 12-fold higher gene

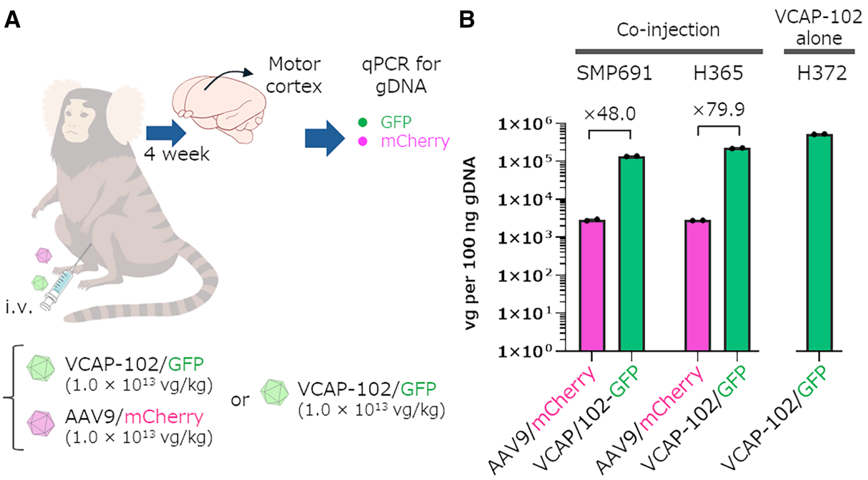

Figure 5. Quantification of vector genome copies in the marmoset brain

(A) Experimental schematic. Two animals (SMP691 and H365) received co-injection of VCAP-102/GFP and AAV9/mCherry at equal doses (1.0×10^{13} vg/kg each), whereas one animal (H372) received VCAP-102/GFP alone (1.0×10^{13} vg/kg). Four weeks after injection, genomic DNA (gDNA) was isolated from the motor cortex and analyzed by qPCR using primers specific for GFP and mCherry. (B) Quantification of vector genome copies detected in cortical gDNA. GFP signals correspond to VCAP-102-derived genomes, and mCherry signals correspond to AAV9-derived genomes. Data are presented as vector genome copies normalized to total gDNA input (vg per 100 ng gDNA), enabling direct comparison across samples.

delivery efficiency than AAV9 based on mRNA quantification,[6] our evaluations showed transduction efficiencies largely comparable to AAV9 under our experimental conditions. In this study, whole-brain fluorescence intensity was used as a readout. While this approach allows for the clear detection of large differences, it may be less sensitive to modest differences of several-fold in viral mRNA expression levels. Therefore, relatively small differences in transduction efficiency may not be readily discernible.

In addition, several factors may account for the discrepancies between our results and previous reports. First, differences in AAV production and recovery methods may influence receptor binding and BBB transport. We used AAV particles naturally released into the serum-free culture supernatant, whereas many prior studies have relied on cell lysis-based recovery. Given that AAV capsids carry glycans and other post-translational modifications (PTMs) and that PTM profiles vary across manufacturing platforms,[16] such differences may affect capsid-endothelial receptor interactions that are critical for efficient BBB penetration.

Second, CPP.16 exhibits concentration-dependent aggregation,[7] and we also observed subtle but clearly detectable aggregates. Even microscale aggregation may impair BBB permeability, and similar effects cannot be excluded for other variants.

Third, genetic differences between marmoset colonies may contribute. As demonstrated by strain-dependent effects of PHP.B between C57BL/6J and BALB/c mice,[3,4,11] differences in genetic background may influence receptor expression or BBB properties.

While whole-brain fluorescence-based evaluation allows robust detection of large differences, it may not resolve modest, several-fold differences in transduction efficiency. Importantly, the primary objective of this study was to identify AAV capsid variants that enable robust and widespread brain transduction following systemic administration, rather than to resolve modest differences in efficiency. For therapeutic applications targeting brain disorders that affect widespread brain regions, such as neurodegenerative diseases, broad and efficient gene delivery across the brain is essential. In this context, relatively small increases in transduction efficiency are unlikely to translate into meaningful therapeutic benefit. Therefore, the use of whole-brain fluorescence intensity provides a practical and efficient strategy to identify variants with clearly superior performance, without requiring genome-level quantification in the initial screening stage.

Taken together, whole-brain fluorescence-based evaluation is well suited to identify capsid variants that achieve robust and widespread brain transduction for therapeutic applications.

Another limitation of the present study is that transduction in peripheral organs was not systematically evaluated. Because the primary aim of this work was to perform a standardized comparison of the BBB penetration efficiency among multiple capsid variants in the marmoset brain, our analyses focused on brain transduction. Future studies assessing systemic biodistribution and off-target transduction will be important for evaluating the safety profile of VCAP-102.

In addition, the present study was conducted in marmosets, which are New World NHPs. Although the marmoset provides a valuable primate model for systematic capsid comparison, further evaluation in Old World NHPs may be important to more fully assess the translational potential of VCAP-102.

Previous studies have reported BBB-penetrant AAV variants with varying performance across species and experimental systems. In contrast, our data demonstrate that VCAP-102 reliably achieves robust BBB penetration and widespread brain transduction in adult marmosets. Importantly, prior work has identified ALPL (tissue-nonspecific alkaline phosphatase) as the receptor for VCAP-102 on brain endothelial cells. Because ALPL is highly conserved across

species from rodents to primates, including humans, this receptor interaction may help explain the strong BBB penetration observed in marmosets. Together, these findings highlight the importance of a systematic evaluation of BBB-penetrant AAV capsids in NHPs and support the potential of VCAP-102 as a promising platform for the development of systemically delivered gene therapies targeting the human brain.

MATERIALS AND METHODS

Animals

The present study included 44 common marmosets (*Callithrix jacchus*). All animals were bred and maintained at the Gunma University Bioresource Center. The marmosets were housed in breeding rooms under controlled environmental conditions, including temperature (27°C–30°C), humidity (25%–45%), and a 12-h light/dark cycle. Filtered water was provided *ad libitum*. The animals were fed 45–50 g of soaked monkey chow (CMS-1; CLEA Japan, Tokyo, Japan) supplemented with fruits, vegetables, soybean flour, quail eggs, or boiled chicken around noon. In addition, "marmoset dumplings" were provided on weekday afternoons around 3:00 p.m. These were prepared by mixing CMS-1 soaked in hot water with honey, oligosaccharides, milk powder, vitamin supplements, *Lactobacillus* powder, and gum arabic powder. All housing and husbandry conditions complied with the Guide for the Care and Use of Laboratory Animals, 8th edition. Every effort was made to minimize animal suffering and to reduce the number of animals used. All procedures for animal care and experimentation were approved by the Japan Neuroscience Society (Guidelines for Experiments on Primates in the Field of Neuroscience) and the Institutional Animal Care and Use Committee of Gunma University (approval nos. 18–019, 20–053, 21–063, 23–018, 23–057, and 24–057).

Construction of plasmids

The expression plasmids pAAV-CBh-GFP-WPRE-hBGpA and pAAV-CBh-mCherry-WPRE-hBGpA were used to drive constitutive expression of GFP or mCherry under the control of the CBh promoter.[17] The Kozak sequence and fluorescent protein genes were inserted immediately downstream of the CBh promoter into the pAAV expression plasmid at the AgeI and NotI restriction sites. The AAV2/9 packaging plasmid (pAAV2/9), which contains the AAV2 *rep* gene and the AAV9 *cap* gene, was provided by James M. Wilson. The cap genes of PHP.B, PHP.eB, AAV-F, CAP-B10, CAP-B22, and VCAP-102 were generated based on AAV9. Humanized DNA sequences corresponding to the amino acid sequences reported in the original publications were incorporated into synthetic oligonucleotides and inserted into appropriate sites of the respective cap genes in pAAV2/9. The AAV-CPP.16 plasmid was kindly provided by Dr. Fengfeng Bei (Harvard Medical School). The plasmid for producing CAP-Mac, pUCmini-iCAP-AAV.CAP-Mac, was kindly provided by Dr. Viviana Gradinaru (California Institute of Technology). The BR1 and BR1N plasmids, both derived from AAV2, were constructed using a similar strategy: the humanized insert sequence was synthesized as an oligonucleotide and subsequently introduced into the target site of pRC2-mi342.[14] All gene engineering procedures were approved by the Institutional Committee of Gunma University (approval nos. 15–038, 20–018, 23–056, 24–054, and 24–070).

CREATE-based screening of AAV capsids

An AAV capsid library was generated using the CREATE method, as previously described.[1] The plasmids rAAV-Cap-in-cis-lox genome, pRep-AAP, and pRep-AAP deltaX/A used for generating the AAV library for CREATE screening were kindly provided by Drs. Viviana Gradinaru and Benjamin Deverman. Unlike in rodents, genetically engineered marmosets that express Cre in specific brain cell types are not readily available. Therefore, Cre expression was achieved using AAV vectors. Specifically, the AAV library was first administered intravenously through the femoral vein. Two weeks after the AAV library injection, a mixture of AAV9/NSE-GFP-P2A-Cre and AAV9/GFAP-GFP-P2A-Cre (mixed in a 1:1 ratio) was injected directly into the cerebral cortex. This order of administration was chosen to avoid the production of anti-AAV9 neutralizing antibodies (NAbs) prior to library delivery. If AAV9 vectors were injected into the brain first, rapid induction of NAbs against AAV9 could be elicited,[18] potentially resulting in capture of all AAV9-based library particles by the NAbs upon subsequent intravenous administration. Three or four weeks after intraparenchymal injection of the Cre-expressing AAV vectors, GFP-positive cortical tissue was dissected under a fluorescence stereomicroscope. AAV genomes were extracted from the collected tissue, and PCR amplification was performed using primers designed to detect Cre-dependent inversion events.

Screening for AAV capsids targeting membrane proteins highly expressed in marmoset brain endothelial cells

To generate stable cell lines expressing the target membrane proteins, the common marmoset (*Callithrix jacchus*) insulin receptor (cjIR) and lymphocyte antigen 6H (cjLy6H) were cloned into the lentiviral expression vector pCL20c/CBh.*-P2A-GFP.WPRE, using PCR and the In-Fusion HD Cloning Kit (TaKaRa Bio, Shiga, Japan). The following primer sets were used: cjIR-F, 5′-TTCAGGTTGGACCGGTGCCACCATGGGAGCCGGGAGCCGCCGG-3′; P2A-cjIR-R, 5′-CGTGGCTCCGGAGCCGGAAGGGTTGGAACGAGGCAAGGCC-3′; cjLy6H-F, 5′-TTCAGGTTGGACCGGTGCCACCATGCTGCCTGCAGCCATGAAGGG-3′; P2A-cjLy6H-R, 5′-CGTGGCTCCGGAGCCGAGCCCCGCCCAGAGGAGGG-3′. VSV-G-pseudotyped lentiviral vectors were produced in HEK293T cells, as previously described,[19,20] and used to establish HEK293T cell lines stably expressing cjIR or cjLy6H on the plasma membrane. Synthetic oligonucleotides containing 8× NNK, corresponding to random 8-amino acid insertions (GenScript, Piscataway, NJ, USA), were inserted into the AAV9 capsid gene to generate a diversified AAV library, which was produced in HEK293T cells. The resulting AAV library was screened using the cjIR- or cjLy6H-expressing stable cell lines. For each target membrane protein, three rounds of screening were performed, and candidate insertion sequences presumed to enhance infection specificity were identified by next-generation sequencing (NGS) analysis. Each candidate was designated AAV9-IR or AAV9-Ly6H, respectively, and their BBB permeability was subsequently examined in marmosets.

Production of AAV vectors

AAV vectors were collected from the culture supernatant. Recombinant single-stranded AAV vectors were produced in HEK293T cells (HCL4517; Thermo Fisher Scientific, Waltham, MA), using the previously described ultracentrifugation method.[21] Briefly, HEK293T cells were cultured in Dulbecco's modified Eagle Medium (DMEM; D5796-500ML, Merck, Darmstadt, Germany) supplemented with 8% fetal bovine serum (26140–079, Sigma-Aldrich) at 37°C in 5% CO_2. The cells were transfected with three plasmids—the pAAV expression plasmid, pHelper (Stratagene, La Jolla, CA), and a rep/cap plasmid—using polyethylenimine "Max" (24765–1; Polysciences, Warrington, PA, USA). Viral particles were harvested from the culture medium 6 days after transfection and concentrated by precipitation with 8% polyethylene glycol 8000 (PEG8000; Merck) and 500 mM sodium chloride. The precipitated vectors were resuspended in D-PBS(−) and purified by iodixanol density gradient ultracentrifugation (OptiPrep; Serumwerk Bernburg AG, Bernburg, Germany). The purified viral solution was then concentrated in D-PBS(−), using a Vivaspin 20 or a Vivaspin Turbo 15 centrifugal concentrator (100,000 MWCO PES; Sartorius, Göttingen, Germany). Genomic titers were determined by quantitative real-time PCR using a Thermal Cycler Dice Real Time System II TP900 or III TP970 (Takara Bio, Shiga, Japan) and Power SYBR Green PCR Master Mix (Thermo Fisher Scientific). The following primers targeting the WPRE sequence were used: 5′-CTGTTGGGCACTGACAATTC-3′ and 5′-GAAGGGACGTAGCAGAAGGA-3′. The expression plasmid was used to generate a standard curve for absolute quantification. AAV preparations were stored at 4°C for short-term use (up to a few months) and at −80°C for long-term storage.

HEK293T cell-based AAV NAb assay

A HEK293T cell-based NAb assay for AAV2 or AAV9 was performed to select marmosets suitable for intravenous AAV administration. Whole blood was collected from candidate animals, and serum was isolated by centrifugation at 5,000 rpm for 5 min at 4°C. Serum samples were stored at 4°C for up to one week or at −80°C for long-term storage. HEK293T cells were seeded at 4×10^4 cells/well in 100 μL of DMEM supplemented with 10% FBS in a 96-well plate (μ-Plate 96 Well Square ibiTreat Sterilized; ibidi, Gräfelfing, Germany) and incubated for 2–6 h at 37°C in 5% CO_2. AAV2/CBh-GFP-WPRE-hBGpA or AAV9/CBh-GFP-WPRE-hBGpA was diluted in serum-free DMEM to achieve optimized multiplicities of infection (MOIs) of 1×10^3 (AAV2) or 5×10^4 (AAV9). Marmoset serum was diluted 1:5 in serum-free DMEM. Equal volumes of diluted serum and titer-adjusted AAV2 or AAV9 were mixed in a 96-well plate (Nunc MicroWell 96-Well; Thermo Fisher Scientific) and incubated for 1 h at 37°C in 5% CO_2. The AAV/serum mixtures were then added to the HEK293T cells and incubated for 72–76 h under the same conditions. GFP fluorescence in HEK293T cells was imaged and quantified using the Image Cytometer Module of the BZ-X800 fluorescence microscope (Keyence, Osaka, Japan). To assess NAb activity, marmoset serum was pre-incubated with AAV2 or AAV9 mutant capsids as positive controls. In addition, samples that were infected with AAV alone, without the incorporation of marmoset serum, served as negative controls. The absence of NAbs was defined as GFP fluorescence ≥50% of the signal in the negative control, which served as the cut-off threshold.

Intravenous injection of AAV vectors

Each AAV vector was administered intravenously into the femoral vein of marmosets. Intravenous injections were performed using one of following two methods: (1) connecting a winged needle and syringe via a three-way stopcock, or (2) directly attaching the syringe to the winged needle, as described previously.[2] After immobilization by intramuscular injection of a mixture of ketamine hydrochloride and xylazine hydrochloride, the AAV vector solution was immediately administered intravenously.

Necropsy and whole-brain fluorescence imaging

Marmosets were anesthetized with a cocktail of ketamine hydrochloride, xylazine hydrochloride, and isoflurane four to five weeks after viral injection (4.6 ± 0.2 weeks, mean ± SEM, n = 32). Animals were perfused transcardially with 300 mL of cold D-PBS(−) (14249–95, Nacalai Tesque, Kyoto, Japan) containing 20 mM EDTA (311–90075, Nippon Gene, Tokyo, Japan), followed by 250 mL of 4% paraformaldehyde (PFA) in 0.1 M phosphate buffer (006775-1L, Bioenno Lifesciences, Santa Ana, CA). Brains were then removed. Following necropsy, GFP and mCherry fluorescence in whole marmoset brains was imaged using a Keyence VB-7010 fluorescence microscope. Fluorescence intensity analysis was performed using Fiji (ImageJ).[22]

Immunohistology

To examine GFP expression in marmoset brain tissue, 100-μm-thick microtome sections were prepared and subjected to fluorescent immunostaining. Brains were bisected medially with a scalpel, the temporal lobes were trimmed, and the remaining tissue was embedded in 2% agarose gel. Sagittal sections (100-μm thick) were obtained using a microtome (VT1200S; Leica Microsystems GmbH, Wetzlar, Germany) and stored at 4°C in 1× PBS(−) containing NaN_3 until use. Sections were stained using triple-fluorescence labeling, including nuclear staining with NucBlue (Hoechst 33342; Thermo Fisher Scientific). Tissue sections were incubated overnight at room temperature with the following primary antibodies diluted in blocking solution (2% donkey serum [S30-100ML; Merck, Darmstadt, Germany], BSA [A9647; Merck], 0.5% Triton X-100, and 0.03% NaN_3 in 1× PB): rat monoclonal anti-GFP antibody (1:1,000; 04404–84; Nacalai Tesque, Kyoto, Japan), rabbit polyclonal anti-DsRed antibody (1:500; 632496, Takara Bio), mouse monoclonal anti-NeuN antibody (1:1,000; MAB377; Merck), mouse monoclonal anti-S100B antibody (1:1,000; S2532, Merck), and goat polyclonal anti-parvalbumin antibody (1:200, PV-Go-Af460, Nittobo medical, Tokyo, Japan). For visualization, the sections were incubated for 3–4 h at room temperature in blocking solution containing the following secondary antibodies and the NucBlue reagent (R37605, Thermo Fisher Scientific): Donkey anti-rat IgG Alexa Fluor Plus 488 (A48269, 1:2,000; Thermo Fisher Scientific), anti-rabbit IgG Alexa Fluor Plus 555 (1:2,000;

A32794, Thermo Fisher Scientific), Donkey anti-mouse IgG Alexa Fluor Plus 647 (1:2,000; A32787, Thermo Fisher Scientific), and anti-goat IgG Alexa Fluor Plus 647 (1:2,000; A32849, Thermo Fisher Scientific). Following secondary antibody incubation, sections were mounted on glass slides using ProLong Glass Antifade Mountant (P36980, Thermo Fisher Scientific), and then allowed to cure at room temperature for three to seven days, and stored at 4°C.

Relative quantification of viral DNA in marmoset brains injected with a single AAV vector

To quantitatively assess the delivery of BBB-permeable AAV vectors to the marmoset brain, the amount of AAV gDNA was measured by qPCR. Marmosets were anesthetized with a cocktail of ketamine hydrochloride, xylazine hydrochloride, and isoflurane six weeks after viral injection (6.6 ± 0.1 week, mean ± SEM, n = 12). Marmosets were perfused with cold D-PBS(−) containing 20 mM EDTA, followed by 1% PFA in 1× phosphate buffer (PB). The left hemisphere was used for histological analysis, whereas the right hemisphere was used for AAV gDNA quantification. Following perfusion fixation, the brains were rapidly removed. The left hemisphere was post-fixed in 4% PFA overnight at 4°C and sectioned into 100-μm-thick slices for immunostaining. Sections were stained with the rat monoclonal anti-GFP antibody (Nacalai Tesque) and Donkey anti-rat IgG Alexa Fluor Plus 488 (Thermo Fisher Scientific). For viral DNA quantification, approximately 25 mg of tissue was dissected from the cerebral cortex and cerebellar cortex of the right hemisphere. Tissue pieces were immersed in 30% ethanol on ice for at least 10 min, followed by immersion in 40% ethanol on ice for at least 10 min. PFA-ethanol displacement was performed by sequential immersion in ethanol solutions of increasing concentration (10% increments), ending with 99.5% ethanol. Ethanol was then allowed to completely evaporate by incubation at 50°C for 1 h. gDNA was subsequently extracted using the Wizard Genomic DNA Purification Kit (Promega, Madison, WI). qPCR quantification was performed using the KAPA SYBR Fast qPCR Kit (Roche, Basel, Switzerland) on the Thermal Cycler Dice Real Time System II TP900 (Takara Bio). Primers were designed to span the GFP and WPRE regions, using the following sequences: qPCR-GFP-For, 5′-GGACGAGCTGTACAAGTAAAG-3′ and qPCR-WPRE-Rev, 5′-GGGAAGCAATAGCATGATACAAAGG-3′. The vg copy numbers were determined by qPCR and are expressed as relative values normalized to AAV9 (set to 1) for each experiment.

Absolute quantification of viral DNA in marmoset brains injected with VCAP-102/GFP and AAV9/mCherry vectors

A comparative analysis of the gDNA copy number in the brain was performed between VCAP-102 and AAV9. Brain tissue was collected from the primary motor cortex of marmosets that had been co-injected with VCAP-102/GFP and AAV9/mCherry, or the VCAP-102/GFP-injected marmoset brain, which had been perfused and fixed with D-PBS followed by 4% PFA. Tissues were immersed in 30% ethanol on ice for at least 10 min, followed by immersion in 40% ethanol on ice for at least 10 min. PFA-ethanol displacement was performed by sequential immersion in ethanol solutions of increasing concentration (10% increments), ending with 99.5% ethanol. Ethanol was then completely evaporated by incubation at 50°C for 1 h. gDNA was extracted from perfusion-fixed brain tissue using the Wizard Genomic DNA Purification Kit (Promega, Madison), as the tissue had already been fixed for histological analysis. qPCR absolute quantification was performed using the Power SYBR Green PCR Master Mix on the Thermal Cycler Dice Real Time System III TP970 (Takara Bio). Standard curves were generated using plasmids containing the GFP or mCherry sequence, and vg copy numbers were calculated based on these curves. The vg copy numbers were normalized to the amount of input gDNA and are presented as vg per 100 ng gDNA. This normalization was used to enable direct comparison of vector delivery across samples under identical experimental conditions. Conversion to vg per cell was not performed due to the heterogeneous cellular composition of brain tissue. The following primer sets were used for qPCR. The GFP and mCherry primer sets were validated to specifically amplify their respective target sequences without cross-amplification: qPCR-GFP-F, 5′-GAAGTTCATCTGCACCACCG-3′; qPCR-GFP-R, 5′-GTCGTGCTGCTTCATGTGGTC-3′; qPCR-mCherry-F, 5′-CCGAGGGCTTCAAGTGGGAG-3′; qPCR-mCherry-R, 5′-CGCAGCTTCACCTTGTAGATG-3′.

Statistical analysis

Statistical analyses and graph generation were performed using GraphPad Prism versions 6 and 10 (GraphPad Software, San Diego, CA). Data comprising multiple groups are presented as scatterplots (Figure 1). Bars indicate mean values, and error bars represent the standard error of the mean (SEM) (Figures 3, S1, and S2). Each dot corresponds to the fluorescence measurement from an individual marmoset brain. Comparisons among multiple groups were performed using the Kruskal-Wallis test followed by Dunn's multiple-comparison test (Figure S1).

DATA AND CODE AVAILABILITY

All data supporting the findings of this study are available within the article and its supplemental information files. Raw data can be provided upon request.

ACKNOWLEDGMENTS

This work was supported by grants from the Program for Brain Mapping by Integrated Neurotechnologies for Disease Studies (Brain/MINDS; JP20dm0207057/JP21dm0207111 [to H.H.]) and Multidisciplinary Frontier Brain and Neuroscience Discoveries (Brain/MINDS 2.0; JP23wm0625001/JP24wm0625103 [to H.H.]) from the Japan Agency for Medical Research and Development (AMED); and by MEXT/JSPS KAKENHI (JP24K15743 [to Y.M.], JP22K06454/JP24H01221 [to A.K.], and JP23H02791 [to H.H.]). The authors thank Asako Ohnishi, Nobue McCullough, Chieko Miyazawa, Ayako Sugimoto, and Keiko Sato for AAV vector production; Motoko Uchiyama, Minako Noguchi, and Yoshiko Nomura for marmoset care and management; Junko Sugi for immunohistochemistry; and Minako Noguchi also for creating the marmoset illustration used in the graphical abstract.

AUTHOR CONTRIBUTIONS

All authors contributed to the overall experimental design. Y.M. managed the raising of marmosets, performed the intravenous AAV injections in marmosets, carried out immunohistochemistry analysis, and conducted data analysis; A.K. and K.S. generated the AAV vectors; A.K. conducted all screening experiments using the AAV capsid variant libraries in both marmosets and cultured cells; Y.U. and T.T. proposed candidate membrane proteins enriched in marmoset brain vascular endothelial cells based on their

own research findings and helped guide the conceptual direction of the study; H.H. supervised the project, provided oversight for data interpretation, edited the manuscript, and finalized the study.

DECLARATION OF INTERESTS

The authors declare no competing interests.

SUPPLEMENTAL INFORMATION

Supplemental information can be found online at https://doi.org/10.1016/j.omta.2026.201783.

REFERENCES

1. Deverman, B.E., Pravdo, P.L., Simpson, B.P., Kumar, S.R., Chan, K.Y., Banerjee, A., Wu, W.L., Yang, B., Huber, N., Pasca, S.P., and Gradinaru, V. (2016). Cre-dependent selection yields AAV variants for widespread gene transfer to the adult brain. Nat. Biotechnol. *34*, 204–209. https://doi.org/10.1038/nbt.3440.
2. Matsuzaki, Y., Konno, A., Mochizuki, R., Shinohara, Y., Nitta, K., Okada, Y., and Hirai, H. (2018). Intravenous administration of the adeno-associated virus-PHP.B capsid fails to upregulate transduction efficiency in the marmoset brain. Neurosci. Lett. *665*, 182–188. https://doi.org/10.1016/j.neulet.2017.11.049.
3. Hordeaux, J., Yuan, Y., Clark, P.M., Wang, Q., Martino, R.A., Sims, J.J., Bell, P., Raymond, A., Stanford, W.L., and Wilson, J.M. (2019). The GPI-Linked Protein LY6A Drives AAV-PHP.B Transport across the Blood-Brain Barrier. Mol. Ther. *27*, 912–921. https://doi.org/10.1016/j.ymthe.2019.02.013.
4. Huang, Q., Chan, K.Y., Tobey, I.G., Chan, Y.A., Poterba, T., Boutros, C.L., Balazs, A.B., Daneman, R., Bloom, J.M., Seed, C., and Deverman, B.E. (2019). Delivering genes across the blood-brain barrier: LY6A, a novel cellular receptor for AAV-PHP.B capsids. PLoS One *14*, e0225206. https://doi.org/10.1371/journal.pone.0225206.
5. Flytzanis, N.C., Goeden, N., Goertsen, D., Cummins, A., Pickel, J., and Gradinaru, V. (2020). Broad gene expression throughout the mouse and marmoset brain after intravenous delivery of engineered AAV capsids. bioRxiv. https://doi.org/10.1101/2020.06.16.152975.
6. Goertsen, D., Flytzanis, N.C., Goeden, N., Chuapoco, M.R., Cummins, A., Chen, Y., Fan, Y., Zhang, Q., Sharma, J., Duan, Y., et al. (2022). AAV capsid variants with brain-wide transgene expression and decreased liver targeting after intravenous delivery in mouse and marmoset. Nat. Neurosci. *25*, 106–115. https://doi.org/10.1038/s41593-021-00969-4.
7. Yao, Y., Wang, J., Liu, Y., Qu, Y., Wang, K., Zhang, Y., Chang, Y., Yang, Z., Wan, J., Liu, J., et al. (2022). Variants of the adeno-associated virus serotype 9 with enhanced penetration of the blood-brain barrier in rodents and primates. Nat. Biomed. Eng. *6*, 1257–1271. https://doi.org/10.1038/s41551-022-00938-7.
8. Chuapoco, M.R., Flytzanis, N.C., Goeden, N., Christopher Octeau, J., Roxas, K.M., Chan, K.Y., Scherrer, J., Winchester, J., Blackburn, R.J., Campos, L.J., et al. (2023). Adeno-associated viral vectors for functional intravenous gene transfer throughout the non-human primate brain. Nat. Nanotechnol. *18*, 1241–1251. https://doi.org/10.1038/s41565-023-01419-x.
9. Moyer, T.C., Hoffman, B.A., Chen, W., Shah, I., Ren, X.-Q., Knox, T., Liu, J., Wang, W., Li, J., Khalid, H., et al. (2024). Highly conserved brain vascular receptor ALPL mediates transport of engineered viral vectors across the blood-brain barrier. bioRxiv. https://doi.org/10.1101/2024.03.12.584703.
10. Moyer, T.C., Hoffman, B.A., Chen, W., Shah, I., Ren, X.Q., Knox, T., Liu, J., Wang, W., Li, J., Khalid, H., et al. (2025). Highly conserved brain vascular receptor ALPL mediates transport of engineered AAV vectors across the blood-brain barrier. Mol. Ther. *33*, 3902–3916. https://doi.org/10.1016/j.ymthe.2025.04.046.
11. Matsuzaki, Y., Tanaka, M., Hakoda, S., Masuda, T., Miyata, R., Konno, A., and Hirai, H. (2019). Neurotropic Properties of AAV-PHP.B Are Shared among Diverse Inbred Strains of Mice. Mol. Ther. *27*, 700–704. https://doi.org/10.1016/j.ymthe.2019.02.016.
12. Chan, K.Y., Jang, M.J., Yoo, B.B., Greenbaum, A., Ravi, N., Wu, W.L., Sánchez-Guardado, L., Lois, C., Mazmanian, S.K., Deverman, B.E., and Gradinaru, V. (2017). Engineered AAVs for efficient noninvasive gene delivery to the central and peripheral nervous systems. Nat. Neurosci. *20*, 1172–1179. https://doi.org/10.1038/nn.4593.
13. Hanlon, K.S., Meltzer, J.C., Buzhdygan, T., Cheng, M.J., Sena-Esteves, M., Bennett, R.E., Sullivan, T.P., Razmpour, R., Gong, Y., Ng, C., et al. (2019). Selection of an Efficient AAV Vector for Robust CNS Transgene Expression. Mol. Ther. Methods Clin. Dev. *15*, 320–332. https://doi.org/10.1016/j.omtm.2019.10.007.
14. Kawabata, H., Konno, A., Matsuzaki, Y., and Hirai, H. (2023). A blood-brain barrier-penetrating AAV2 mutant created by a brain microvasculature endothelial cell-targeted AAV2 variant. Mol. Ther. Methods Clin. Dev. *29*, 81–92. https://doi.org/10.1016/j.omtm.2023.02.016.
15. Hoshi, Y., Uchida, Y., Tachikawa, M., Inoue, T., Ohtsuki, S., and Terasaki, T. (2013). Quantitative atlas of blood-brain barrier transporters, receptors, and tight junction proteins in rats and common marmoset. J. Pharm. Sci. *102*, 3343–3355. https://doi.org/10.1002/jps.23575.
16. Rumachik, N.G., Malaker, S.A., Poweleit, N., Maynard, L.H., Adams, C.M., Leib, R.D., Cirolia, G., Thomas, D., Stamnes, S., Holt, K., et al. (2020). Methods Matter: Standard Production Platforms for Recombinant AAV Produce Chemically and Functionally Distinct Vectors. Mol. Ther. Methods Clin. Dev. *18*, 98–118. https://doi.org/10.1016/j.omtm.2020.05.018.
17. Gray, S.J., Foti, S.B., Schwartz, J.W., Bachaboina, L., Taylor-Blake, B., Coleman, J., Ehlers, M.D., Zylka, M.J., McCown, T.J., and Samulski, R.J. (2011). Optimizing promoters for recombinant adeno-associated virus-mediated gene expression in the peripheral and central nervous system using self-complementary vectors. Hum. Gene Ther. *22*, 1143–1153. https://doi.org/10.1089/hum.2010.245.
18. Shinohara, Y., Konno, A., Nitta, K., Matsuzaki, Y., Yasui, H., Suwa, J., Hiromura, K., and Hirai, H. (2019). Effects of Neutralizing Antibody Production on AAV-PHP.B-Mediated Transduction of the Mouse Central Nervous System. Mol. Neurobiol. *56*, 4203–4214. https://doi.org/10.1007/s12035-018-1366-4.
19. Matsuzaki, Y., Oue, M., and Hirai, H. (2014). Generation of a neurodegenerative disease mouse model using lentiviral vectors carrying an enhanced synapsin I promoter. J. Neurosci. Methods *223*, 133–143. https://doi.org/10.1016/j.jneumeth.2013.12.004.
20. Torashima, T., Koyama, C., Iizuka, A., Mitsumura, K., Takayama, K., Yanagi, S., Oue, M., Yamaguchi, H., and Hirai, H. (2008). Lentivector-mediated rescue from cerebellar ataxia in a mouse model of spinocerebellar ataxia. EMBO Rep. *9*, 393–399. https://doi.org/10.1038/embor.2008.31.
21. Konno, A., and Hirai, H. (2020). Efficient whole brain transduction by systemic infusion of minimally purified AAV-PHP.eB. J. Neurosci. Methods *346*, 108914. https://doi.org/10.1016/j.jneumeth.2020.108914.
22. Schindelin, J., Arganda-Carreras, I., Frise, E., Kaynig, V., Longair, M., Pietzsch, T., Preibisch, S., Rueden, C., Saalfeld, S., Schmid, B., et al. (2012). Fiji: an open-source platform for biological-image analysis. Nat. Methods *9*, 676–682. https://doi.org/10.1038/nmeth.2019.

OMTA, Volume *34*

Supplemental information

Systematic evaluation of BBB-penetrant AAV capsids in marmosets identifies VCAP-102 as a highly efficient brain-transducing capsid

Yasunori Matsuzaki, Ayumu Konno, Kenji Sakamoto, Yasuo Uchida, Tetsuya Terasaki, and Hirokazu Hirai

Figure S1.

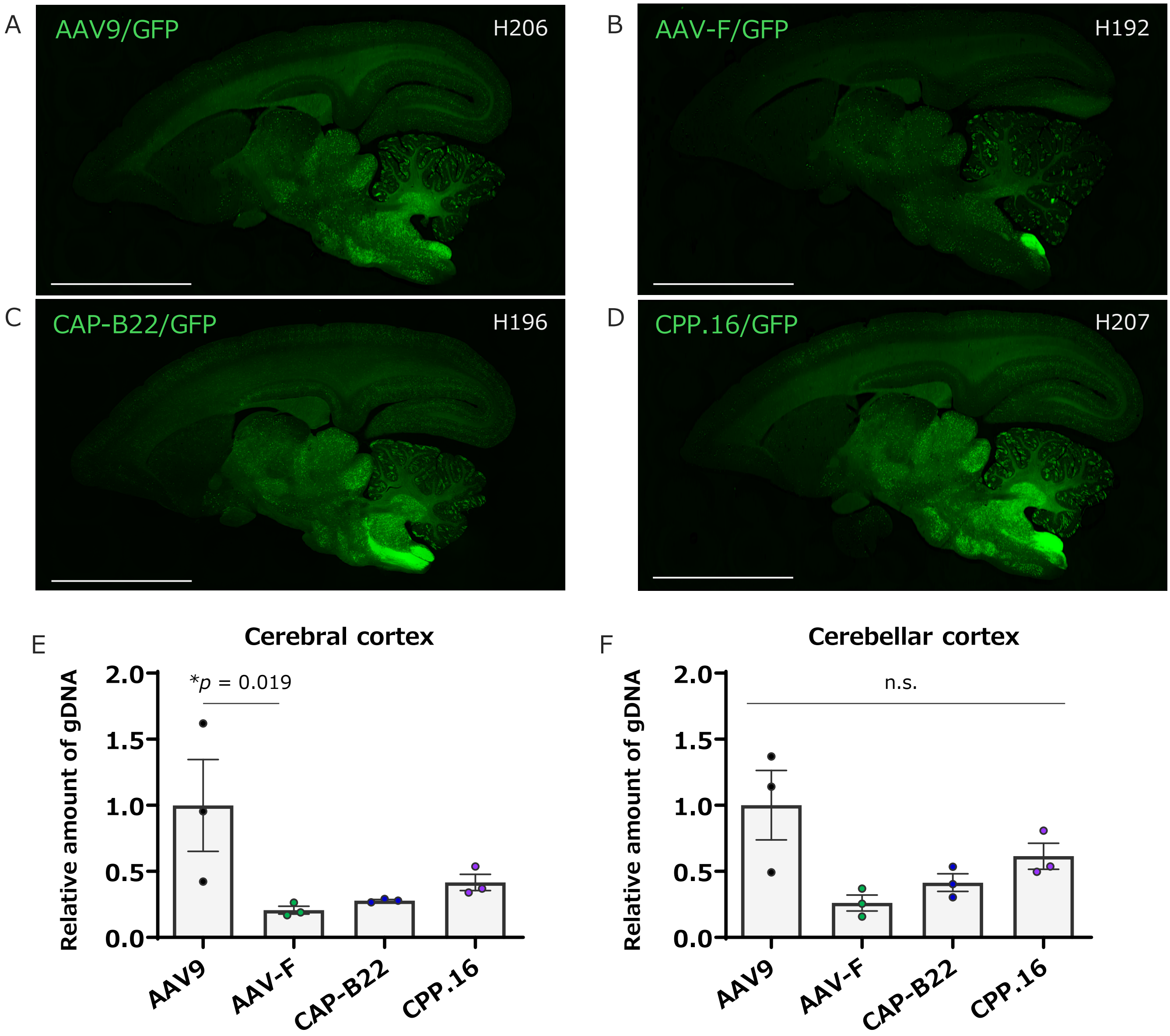

Figure S1. Quantification of AAV genome DNA levels in the marmoset brain following intravenous injection of AAV-F, CAP-B22, or CPP.16.

(A–D) Representative coronal brain images from marmosets injected with AAV9-GFP (A), AAV-F-GFP (B), CAP-B22-GFP (C), or CPP.16-GFP (D). Scale bars; 10 mm.

(E, F) Quantification of AAV genomic DNA (gDNA) in the cerebral cortex (E) and cerebellar cortex (F) by qPCR. gDNA levels for each capsid variant were normalized to those of AAV9. Consistent with the results described in the main text, none of the three variants (AAV-F, CAP-B22, CPP.16) exceeded AAV9 in cortical gDNA levels, and AAV-F showed significantly lower levels than AAV9 in the cerebral cortex ($p = 0.019$). No significant differences were observed among capsids in the cerebellar cortex. Error bars indicate the SEM, and each dot represents the value obtained from an individual marmoset.

Figure S2.

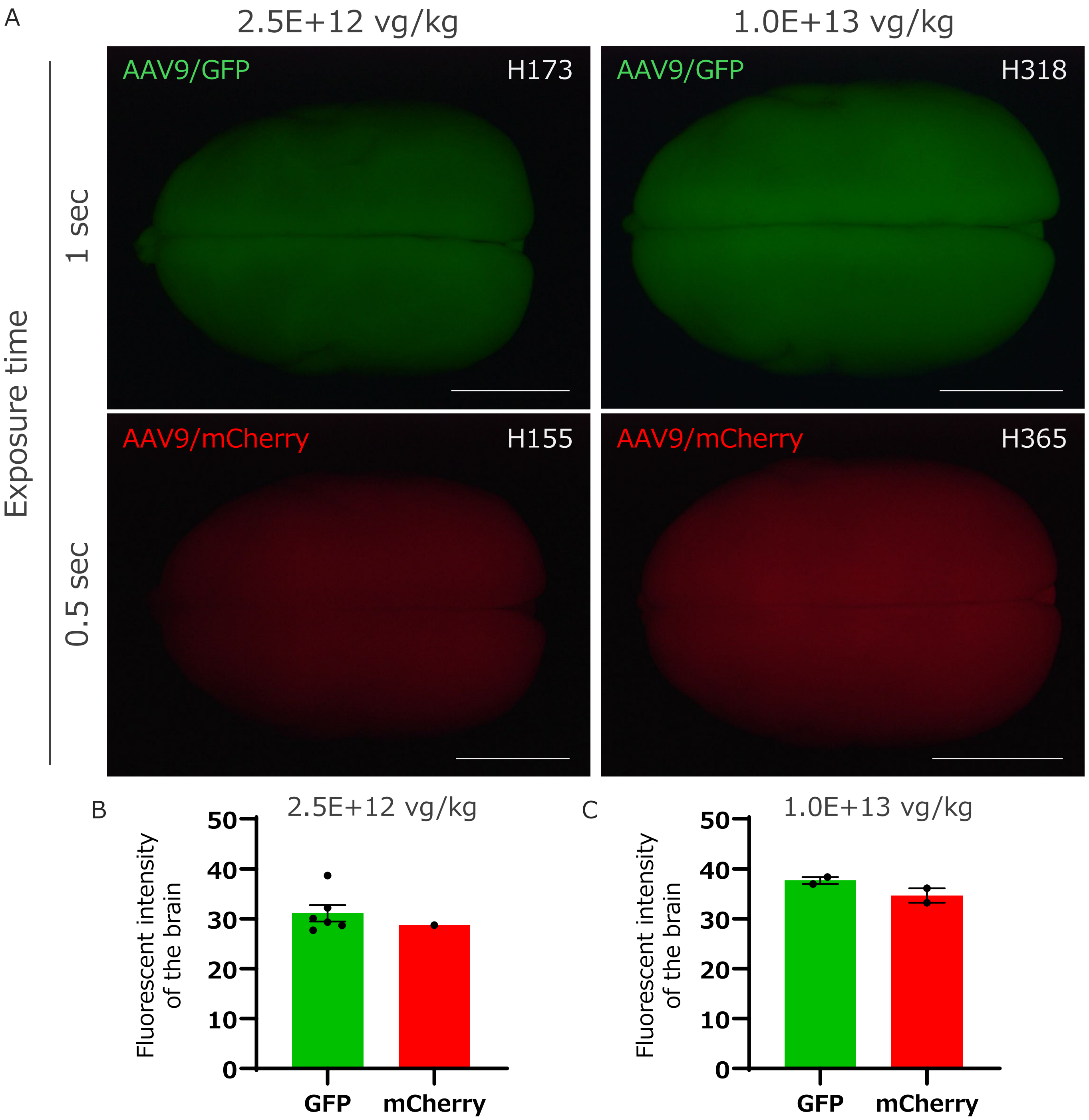

Figure S2. Comparison of whole-brain fluorescence intensity between AAV9-GFP and AAV9-mCherry at different exposure times.

(A) Whole-brain fluorescence images of marmosets intravenously injected with AAV9-GFP (1 s exposure) or AAV9-mCherry (0.5 s exposure) at doses of 2.5×10^{12} vg/kg (left) or 1.0×10^{13} vg/kg (right). Scale bars; 10 mm.

(B, C) Quantification of brain fluorescence intensity at each dose. AAV9-GFP (1 s exposure) and AAV9-mCherry (0.5 s exposure) showed comparable whole-brain fluorescence levels. Therefore, GFP and mCherry signals were imaged using 1 s and 0.5 s exposure times, respectively, throughout this study.

Figure S3.

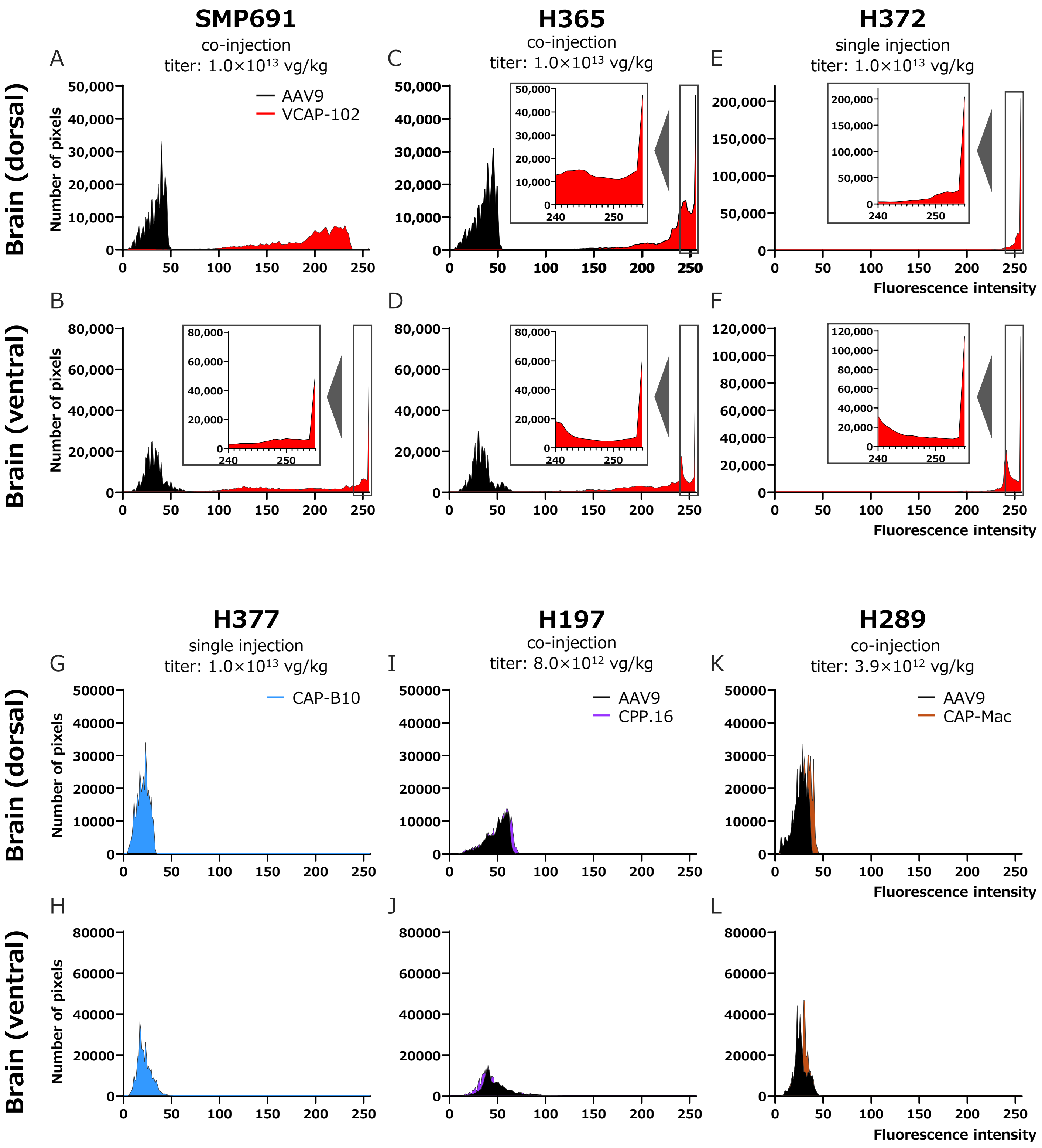

SMP691
co-injection
titer: 1.0×10^13 vg/kg
H365
co-injection
titer: 1.0×10^13 vg/kg
H372
single injection
titer: 1.0×10^13 vg/kg
A
B
C
D
E
F
Brain (dorsal)
Brain (ventral)
Number of pixels
AAV9
VCAP-102
Fluorescence intensity
H377
single injection
titer: 1.0×10^13 vg/kg
H197
co-injection
titer: 8.0×10^12 vg/kg
H289
co-injection
titer: 3.9×10^12 vg/kg
G
H
I
J
K
L
CAP-B10
AAV9
CPP.16
AAV9
CAP-Mac

Figure S3. Whole-brain fluorescence intensity histograms of AAV-injected marmosets.
Whole-brain dorsal (A, C, E, G, I, K) and ventral (B, D, F, H, J, L) fluorescence intensity histograms obtained three to four weeks after intravenous AAV administration in six marmosets (SMP691, H289, H365, H372, H377, H197). AAV9, VCAP-102, CAP-B10, CPP.16, and CAP-Mac were evaluated either by single injection or by co-injection, as indicated for each animal. Fluorescence intensity was recorded as relative values ranging from 0 to 255. For capsids other than VCAP-102, exposure times were minimized to the detection threshold (GFP: 1 s, mCherry: 0.5 s).
Insets in panels B–F show enlarged views of the upper-intensity range (240–255), highlighting the distribution of pixels near the saturation level. Because saturated pixels were recorded as 255, all brain regions in which VCAP-102 expression exceeded the dynamic range of detection appear as a vertical accumulation at this value.
Despite using shortened exposure conditions, all three marmosets injected with VCAP-102 exhibited strong GFP expression with signals saturating the upper limit (255 a.u.) across multiple brain regions. As saturated values were stored as 255 in the histogram analysis, the actual fluorescence intensity of VCAP-102 relative to AAV9 is likely higher than the ratios shown in Figure 3.

Figure S4.

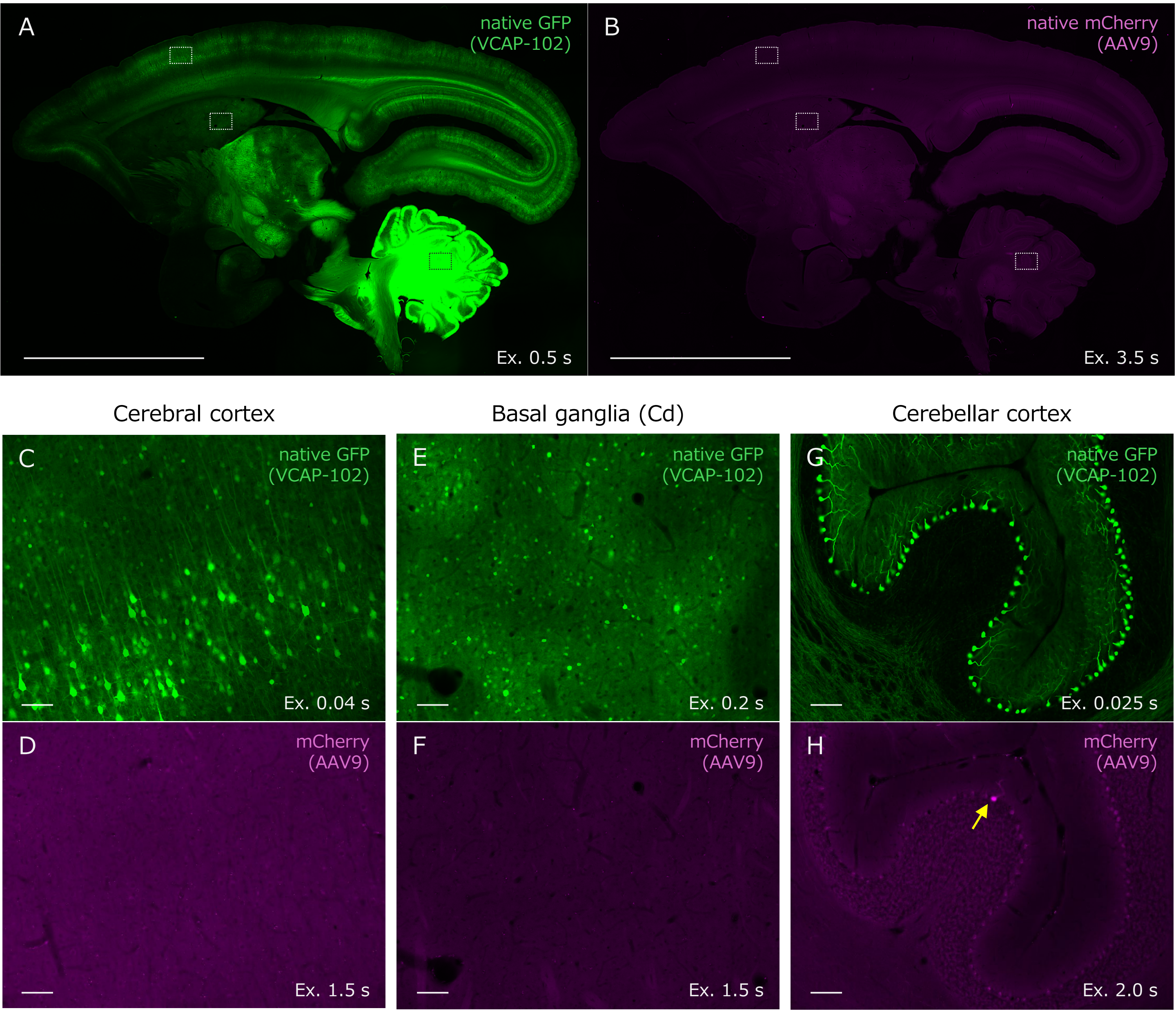

Figure S4. Intravenous administration of VCAP-102 induces widespread GFP expression throughout the brain.
(A, B) Representative sagittal brain images showing native GFP (A) and mCherry (B) fluorescence in marmoset H365 co-injected with VCAP-102-GFP and AAV9-mCherry. GFP images were acquired with an exposure time of 0.5 s (Ex. 0.5 s). Because mCherry fluorescence was much weaker, the image was acquired with a longer exposure time of 3.5 s (Ex. 3.5 s). Scale bars, 10 mm.
(C–H) Higher-magnification images of the cerebral cortex (C, D), caudate nucleus (E, F), and cerebellar cortex (G, H). Native GFP (upper panels) and mCherry (lower panels) fluorescence images are shown. These images correspond to the boxed regions in the whole sagittal sections shown in (A, B) and were acquired at the exposure times indicated in the lower right corner of each panel. GFP signals were readily detected in numerous cells across all regions, whereas mCherry signals were barely detectable even with substantially longer exposure times. Only a single labeled cell was observed in the cerebellum (arrow in panel H). Scale bars, 100 µm.

Figure S5.

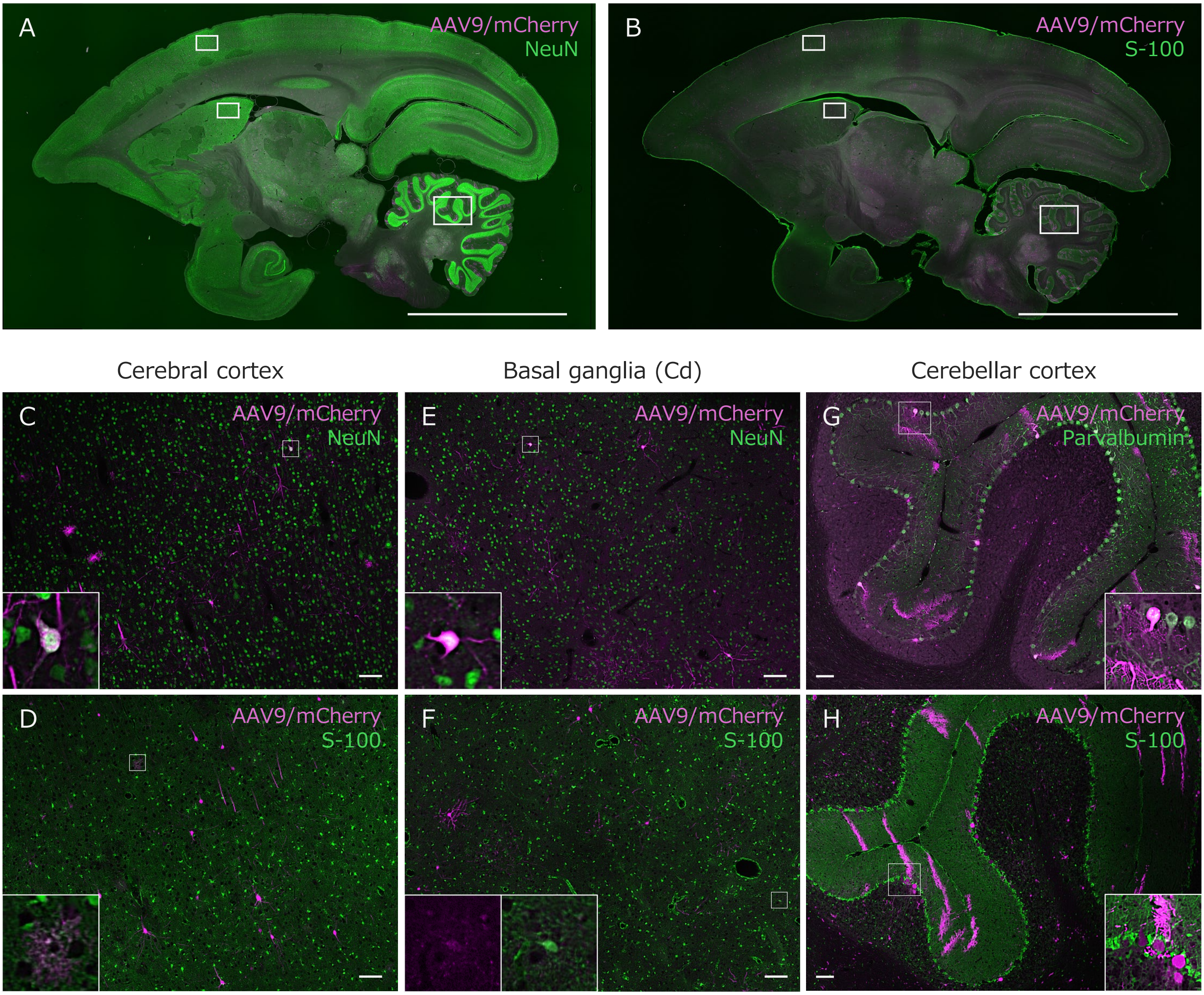

Figure S5. Representative immunohistochemical images of the brain from a marmoset intravenously injected with AAV9-mCherry.

(A, B) Immunohistochemistry of whole-brain sagittal sections showing mCherry/NeuN (A) and mCherry/S100β (B) signals in marmoset H365 injected with AAV9-mCherry. Brain sections were immunolabeled for mCherry together with NeuN (neuron marker), S100β (astrocyte marker), or parvalbumin (PV; inhibitory neuron marker). Scale bars, 10 mm.

(C–H) Higher-magnification images of the cerebral cortex (C, D), caudate nucleus (Cd; E, F), and cerebellar cortex (G, H). Panels show mCherry/NeuN (C, E), mCherry/PV (G), and mCherry/S100β (D, F, H) immunostaining. These images correspond to the boxed regions in (A, B). Insets in the lower left of each panel show further magnified views. Scale bars, 10 mm (A, B) and 100 μm (C-H).

Table S1. Summary of wild-type AAV serotypes and BBB-penetrant AAV capsid variants (2016–2025).

Name of AAV vector	AA452–458 (VR-IV)	AA587–590 (VR-VIII)	Proposal receptor	Method	Publication year
AAV9 (AAV hu.14)	NGSGQNQ	AQAQ	-	-	2004
AAV-PHP.B	NGSGQNQ	AQTLAVPFKAQ	Ly6A (mouse only)	CREATE	2016
AAV-PHP.eB	NGSGQNQ	DGTLAVPFKAQ	Ly6A (mouse only)	CREATE	2017
AAV-F	NGSGQNQ	AQFVVGQSYAQ	Ly6C1 (mouse only)	iTransduce	2019
AAV.CAP-B10	DGAATKN	DGTLAVPFKAQ	(unknown)	M-CREATE	2022
AAV.CAP-B22	DGQSSKS	DGTLAVPFKAQ	(unknown)	M-CREATE	2022
AAV.CPP.16	NGSGQNQ	AQTVSALKAQ	(transcytosis by the cpp)	CPPs-insertion	2022
AAV.CAP-Mac	NGSGQNQ	AQLNTTKPIAQ	(unknown)	*in vivo* directed evolution	2023
AAV-IR	NGSGQNQ	AQGGGSSRHDNGTKGGSGSAQ	(unknown)	TRADE	2024
AAV-Ly6H	NGSGQNQ	AQGGGSNYTKEAMSGGGGSAQ	(unknown)	TRADE	2024
VCAP-102	NGHDSPHKSGQNQ	AQAQ	ALPL/TNAP	TRACER	2025
AAV2	SGTTTQS	NRQA	-	-	1996
AAV-BR1	SGTTTQS	QRGNRGTEWDAQA	(unknown)	random AAV display peptide libraries	2016
AAV-BR1N	SGTTTQS	NRGNRGTEWDAQA	(unknown)	BR1 variant (587N)	2023

This table lists the amino acid sequences inserted into variable region IV (VR-IV; aa452–458 in AAV9) and variable region VIII (VR-VIII; aa587–590 in AAV9), along with proposed or validated endothelial receptors, engineering strategies, and publication years for each capsid. AAV9 (published in 2004) and AAV2 (1996) represent wild-type serotypes. BBB-penetrant AAV capsid variants have been reported from 2016 to 2025. BR1 is not a BBB-penetrant variant but an AAV2-based capsid that efficiently transduces brain endothelial cells; replacement of residue 587 (Q→N) generates BR1N, which acquires BBB permeability in mice.

Amino acids 452–458 are located within the VR-IV region, and amino acids 587–590 are located within the VR-VIII region. Receptor information is provided only when experimentally demonstrated or explicitly proposed in the original studies. “Method” indicates the engineering strategy used (e.g., CREATE, M-CREATE, TRACER, CPP insertion, or directed evolution). Among the listed variants, VCAP-102 is the only capsid with a confirmed endothelial receptor (ALPL/TNAP) in non-human primates.

Table S2. Individual marmoset information and whole-brain dorsal and ventral fluorescence intensities following intravenous AAV administration.

Titer (vg/kg)	Individual ID	Name	AAV capsid	Fluorescent protein	Mean fluorescence intensity dorsal/ventral (a.u., 0–255)
1.0×10^{12}	H193	Ginga	AAV9	GFP	42.2 / 40.8
			CPP.16	mCherry	51.1 / 61.1
2.5×10^{12}	H165	Nogi	AAV9	GFP	27.5 / 29.4
			PHP.eB	mCherry	28.8 / 28.4
	H184	Tanpopo	AAV9	GFP	33.5 / 27.5
			PHP.eB	mCherry	32.0 / 24.2
	H173	Waboku	AAV9	GFP	28.6 / 29.7
			AAV-F	mCherry	28.4 / 22.6
	H186	Fuuwa	AAV9	GFP	30.1 / 32.8
			CAP-B10	mCherry	31.8 / 29.5
	H187	Kaoru	AAV9	GFP	38.6 / 36.3
			CAP-B22	mCherry	29.4 / 27.6
	H183	Uguisu	AAV9	GFP	30.1 / 31.6
			CAP-B22	mCherry	28.9 / 26.2
	H154	Edamame	AAV9	GFP	29.3 / 31.9
			CPP.16	mCherry	28.9 / 27.0
	H155	Okura	AAV9	mCherry	27.7 / 27.3
			CPP.16	GFP	34.5 / 39.0
	H188	Umi	AAV9	GFP	32.2 / 33.9
			BR1N	mCherry	29.3 / 25.6
3.9×10^{12}	H289	Yoru	AAV9	GFP	25.3 / 26.1
			CAP-Mac	mCherry	30.0 / 27.7
5.0×10^{12}	H057	Kasumi	AAV9	GFP	23.2 / 22.7
	I4471	Ren	AAV9	GFP	16.2 / 19.0
	H058	Mitsuba	PHP.B	GFP	22.4 / 26.2
	I4466	Suigetsu	PHP.B	GFP	20.4 / 22.4
8.0×10^{12}	H197	Kuri	AAV9	GFP	47.9 / 50.1
			CPP.16	mCherry	49.9 / 44.4
1.0×10^{13}	H137	Touga	PHP.eB	GFP	18.7 / 25.1
	H148	Tougetsu	PHP.eB	GFP	16.5 / 21.5
	H377	Bon	CAP-B10	GFP	19.4 / 21.1
	H318	Serori	AAV9	GFP	36.9 / 39.2
			AAV-IR	mCherry	32.1 / 29.4
	H319	Paseri	AAV9	GFP	38.4 / 42.0
			AAV-Ly6H	mCherry	44.8 / 43.1
	SMP691	Kinmokusei	AAV9	mCherry	33.2 / 32.1
			VCAP-102	GFP	188.8 / 187.1
	H365	Nodoka	AAV9	mCherry	36.1 / 32.8
			VCAP-102	GFP	229.4 / 213.2
	H372	Nana	VCAP-102	GFP	248.6 / 241.4
2.0×10^{13}	H140	Sekka	AAV9	GFP	21.6 / 22.6
	H141	Neyuki	PHP.eB	GFP	35.9 / 41.3

Mean pixel intensity (0–255 a.u.) was quantified on the dorsal ventral surface of the whole brain approximately 4 weeks after intravenous delivery of each AAV vector. For animals receiving co-injections, fluorescence from AAV9 (GFP or mCherry) serves as the internal control for the paired variant. The table lists, for each individual: AAV dose (vg/kg), animal ID, name, capsid, expressed fluorophore, and mean dorsal and ventral fluorescence intensity. Measurements were performed under standardized exposure settings (GFP: 1 s; mCherry: 0.5 s).
